# A multimodal fusion system predicting survival benefits of immune checkpoint inhibitors in unresectable hepatocellular carcinoma

**DOI:** 10.1038/s41698-025-00979-6

**Published:** 2025-06-14

**Authors:** Jun Xu, Tengfei Wang, Junjun Li, Yong Wang, Zhangxiang Zhu, Xiao Fu, Junjie Wang, Zhenglin Zhang, Wei Cai, Ruipeng Song, Changlong Hou, Li-Zhuang Yang, Hongzhi Wang, Stephen T. C. Wong, Hai Li

**Affiliations:** 1 Hefei Cancer Hospital of CAS, Institute of Health and Medical Technology, Hefei Institutes of Physical Science, Chinese Academy of Sciences, Hefei, P. R. China; 2 University of Science and Technology of China, Hefei, P. R. China; 3 Department of Oncology, Hefei Cancer Hospital; Chinese Academy of Sciences, Hefei, P. R. China; 4 Department of Interventional Radiology, The First Affiliated Hospital of USTC, Division of Life Sciences and Medicine, University of Science and Technology of China, Hefei, P. R. China; 5 Department of Radiology, The First Affiliated Hospital of University of Science and Technology of China, Hefei, P. R. China; 6 Department of Radiology, The First Affiliated Hospital of Wannan Medical College (Yijishan Hospital of Wannan Medical College), Wuhu, P. R. China; 7 Department of Radiology, The First Affiliated Hospital of Anhui Medical University, Hefei, P. R. China; 8 Department of Hepatobiliary Surgery, The First Affiliated Hospital of USTC, Division of Life Sciences and Medicine, the University of Science and Technology of China, Anhui Province Key Laboratory of Hepatopancreatobiliary Surgery, Anhui Provincial Clinical Research Center for Hepatobiliary Diseases, Hefei, P. R. China; 9 Department of Systems Medicine and Bioengineering, Houston Methodist Neal Cancer Center, Houston Methodist Hospital, Houston, TX USA; 10 Department of Radiology, Weill Cornell Medical College, New York, NY USA

**Keywords:** Prognostic markers, Tumour biomarkers, Hepatocellular carcinoma, Cancer imaging, Risk factors, Outcomes research

## Abstract

Early identification of unresectable hepatocellular carcinoma (HCC) patients who may benefit from immune checkpoint inhibitors (ICIs) is crucial for optimizing outcomes. Here, we developed a multimodal fusion (MMF) system integrating CT-derived deep learning features and clinical data to predict overall survival (OS) and progression-free survival (PFS). Using retrospective multicenter data (*n* = 859), the MMF combining an ensemble deep learning (Ensemble-DL) model with clinical variables achieved strong external validation performance (C-index: OS = 0.74, PFS = 0.69), outperforming radiomics (29.8% OS improvement), mRECIST (27.6% OS improvement), clinical benchmarks (C-index: OS = 0.67, *p* = 0.0011; PFS = 0.65, *p* = 0.033), and Ensemble-DL (C-index: OS = 0.69, *p* = 0.0028; PFS = 0.66, *p* = 0.044). The MMF system effectively stratified patients across clinical subgroups and demonstrated interpretability through activation maps and radiomic correlations. Differential gene expression analysis revealed enrichment of the PI3K/Akt pathway in patients identified by the MMF system. The MMF system provides an interpretable, clinically applicable approach to guide personalized ICI treatment in unresectable HCC.

## Introduction

Hepatocellular carcinoma (HCC) is a highly aggressive malignancy, with over 70% of patients diagnosed at advanced stages, rendering them ineligible for surgical resection, resulting in a dismal prognosis^1,2^. While immune checkpoint inhibitors (ICIs) combined with targeted therapies, such as anti-angiogenic drugs or tyrosine kinase inhibitors (TKIs), have significantly improved HCC treatment, response rates remain limited at around 30%^3,4^. This highlights the need for improved strategies to optimize immunotherapy for patients with HCC, in particular, the development of effective biomarkers to pre-screen potential patients who are sensitive to immunotherapy, thereby increasing the effectiveness of immunotherapy.

Currently, there are still no universally accepted biomarkers that can accurately predict the efficacy of immunotherapy in HCC. Biomarkers such as PD-L1 expression on tumor and immune cells, tumor mutational burden (TMB), and microsatellite instability (MSI) are used in clinical practice to identify patients with various malignancies likely to respond to ICIs^5–7^. However, their clinical utility in HCC is limited due to pronounced immune heterogeneity and low incidence rates of these biomarkers in HCC^8–10^. Contrast-enhanced CT is also widely used for HCC diagnosis and therapy assessment, providing comprehensive information about the tumor and its surroundings^11^. Based on CT imaging, the modified Response Evaluation Criteria in Solid Tumors (mRECIST) is the standard tool for assessing response in HCC clinical trials^12^. However, immunotherapy often induces unconventional response patterns that are not adequately captured by size-based criteria, such as mRECIST^13^.

Advancements in artificial intelligence (AI), especially deep learning, are transforming radiology^14,15^. Deep learning employs complex neural networks to analyze and quantify imaging data, extracting features that reveal underlying disease states often invisible to the naked eye. Unlike handcrafted radiomics with predefined features, deep learning identifies or creates more effective “deep features”, enabling a comprehensive assessment of tumor heterogeneity non-invasively^16,17^. Several studies have explored developing radiomics or deep learning biomarkers to predict immunotherapy outcomes in HCC, showing promising clinical potential^18^. However, these investigations encounter several limitations^11,19–23^. First, most research primarily focuses on evaluating the short-term efficacy of immunotherapy in HCC patients using criteria such as mRECIST. However, there is limited emphasis on predicting long-term efficacy, including overall survival (OS) and progression-free survival (PFS). Predictive biomarkers for assessing long-term efficacy hold significant clinical value, as they can provide physicians with comprehensive information to assist in making reliable long-term treatment decisions^24^. Second, current deep learning research mainly relies on a single network architecture, which struggles to handle the complex characteristics of the tumor microenvironment, resulting in prediction models that often suffer from problems such as overfitting and instability. In contrast, an ensemble model incorporating multiple single models can address the shortcomings of traditional methods by integrating complementary information and multidimensional data, thereby improving the accuracy and stability of prediction models. Third, lack of interpretability and black-box characteristics are another major limitation of AI-generated imaging biomarkers, which limits their wide clinical application. Combining radiomics with gene expression analysis can provide insights into the relationship between imaging and genomic data, helping us understand the biological mechanisms underlying these biomarkers. Finally, current deep learning studies for HCC typically involve limited sample sizes (usually less than 220 patients) due to the scarcity of data, especially the difficulty in obtaining data OS and PFS, which raises concerns about the generalisability of the findings^11,18,19^. Small sample sizes may hinder the applicability of models to larger patient populations.

This study developed and validated an innovative multimodal fusion (MMF) system to assess survival benefits for patients with unresectable HCC receiving various ICIs treatment regimens. The MMF system, based on an ensemble learning strategy, overcame the limitations of traditional methods by integrating a deep learning signature from multimodal CT imaging with key clinical features. This approach enhanced understanding of the tumor microenvironment and improved the accuracy and robustness of prognostic predictions. Furthermore, the study analyzed the complementary roles of deep learning-derived CT features and clinical features, exploring their correlations with interpretable radiomics features and gene expression to optimize the MMF system and reveal potential biological mechanisms. Validated on external cohorts from three hospitals, the MMF system effectively identified patients likely to benefit from ICIs therapy, thereby improving survival outcomes and providing valuable support for clinical decision-making.

## Results

### Study patient characteristics

Table 1 summarizes the baseline characteristics of the training (*n* = 520), validation (*n* = 130), and external testing (*n* = 209) cohorts. Among the 859 included patients, the median age was 58 years (IQR, 51–67 years), with 736 males and 123 females. 84.3% (724/859) were infected with hepatitis B virus (HBV), and 75.3% (647/859) had liver cirrhosis. First-line ICIs therapy was administered to 86.0% (739/859) of patients, and 34.2% (294/859) had prior local therapy. No significant differences in patient parameters were observed across the three cohorts. Additionally, the Cancer Imaging Archive (TCIA) cohort included 46 patients with a median age of 65(IQR, 54–68 years; 29 males and 17 females).

**Table 1:** Characteristics of the training cohort, validation cohort and testing cohort

Characteristic	Training cohort (*n* = 520)	Validation cohort (*n* = 130)	*p* value^*^	Testing cohort (*n* = 209)	*p* value^*^
**Age, years**	58 [51–67]	57 [49-65]	0.070	58 [52-67]	0.323
**Sex (%)**			0.174		0.519
Male	439 (84.4)	114 (87.7)		183 (87.6)	
Female	81 (15.6)	16 (12.3)		26 (12.4)	
**Etiology**			0.356		0.419
Hepatitis B virus	431 (82.9)	109 (83.8)		184 (88.0)	
Others	89 (17.1)	21 (16.2)		25 (12.0)	
**Cirrhosis**			0.435		0.667
Yes	387 (74.4)	100 (76.9)		160 (76.6)	
No	133 (25.6)	30 (23.1)		49 (23.4)	
**Child-Pugh class**			0.383		0.419
A	433 (83.2)	103 (79.2)		184 (88.0)	
B	87 (16.7)	27 (20.8)		25 (12.0)	
**ECOG PS**			0.877		0.274
0	298 (57.3)	69 (53.1)		128 (61.2)	
1	222 (42.7)	61 (46.9)		81 (38.2)	
**BCLC stage**			0.486		1.000
B	45 (8.7)	14 (10.2)		26 (12.4)	
C	475 (91.3)	116 (89.2)		183 (87.6)	
**AFP level (ng/mL)**			0.089		0.445
≤400	315 (60.6)	55 (57.7)		124 (59.3)	
>400	205 (39.4)	75 (42.3)		85 (40.7)	
**Up-to-seven criteria**			0.489		0.832
≤7	227 (43.7)	63 (48.5)		102 (48.8)	
>7	293 (56.3)	67 (51.5)		107 (51.2)	
**Tumor size (cm)**	8.7 [5.5-12.0]	8.7 [5.6-12.0]	0.950	8.0 [5.1-11.4]	0.084
**Portal vein invasion**			0.220		0.829
Absent	84 (16.2)	21 (16.2)		42 (20.1)	
Present	436 (83.8)	109 (83.8)		167 (79.9)	
**Lung metastasis**			1.000		0.592
Absent	404 (77.7)	100 (76.9)		173 (82.8)	
Present	116 (22.3)	30 (23.1)		36 (17.2)	
**Bone metastasis**			1.000		1.000
Absent	498 (95.8)	124 (95.4)		207 (99.0)	
Present	22 (4.2)	6 (4.6)		2 (1.0)	
**Lymph node metastasis**			1.000		0.728
Absent	364 (70.0)	91 (70.0)		140 (67.0)	
Present	156 (30.0)	39 (30.0)		69 (33.0)	
**Line of ICIs therapy (n, %)**			0.909		0.845
1	456 (87.7)	110 (84.6)		173 (82.8)	
≥2	64 (12.3)	20 (15.3)		36 (17.2)	
**Prior local therapy (n, %)**			0.252		0.128
Absent	341 (65.6)	78 (60.0)		146 (69.9)	
Present	179 (34.4)	52 (40.0)		63 (30.1)	
**Duration of follow up (months)**	22.92 [15.55-31.76]	22.98 [14.79-30.12]	0.944	25.81 [21.67-32.32]	0.035
**Median OS (months)**	18.48 [9.76-37.87]	18.48 [8.52-41.03]	0.685	21.28 [10.62-40.51]	0.126
**Median PFS(months)**	7.53 [3.60-16.54]	7.15 [3.19-19.08]	0.858	8.17 [4.37-18.97]	0.213

Data are median (interquartile range) or n (%). Up-to-seven criteria, an assessment metric of tumor burden, defined as the sum of tumor count and maximum tumor diameter (cm), with a threshold ≤7.

*Indicates the comparison to the training cohort. Mann–Whitney U and the Student’s t test for continuous variables and Chi-squared test or Fisher exact test for categorical variables were applied.

*ECOG PS* Eastern Cooperative Oncology Group performance status, *BCLC* Barcelona Clinic Liver Cancer, *AFP* alpha fetoprotein, *ICIs* immune checkpoint inhibitor, *HCC* hepatocellular carcinoma, *OS* overall survival, *PFS* progression-free survival.

### Performance of MMF system for survival prediction

**Fig. 1:**
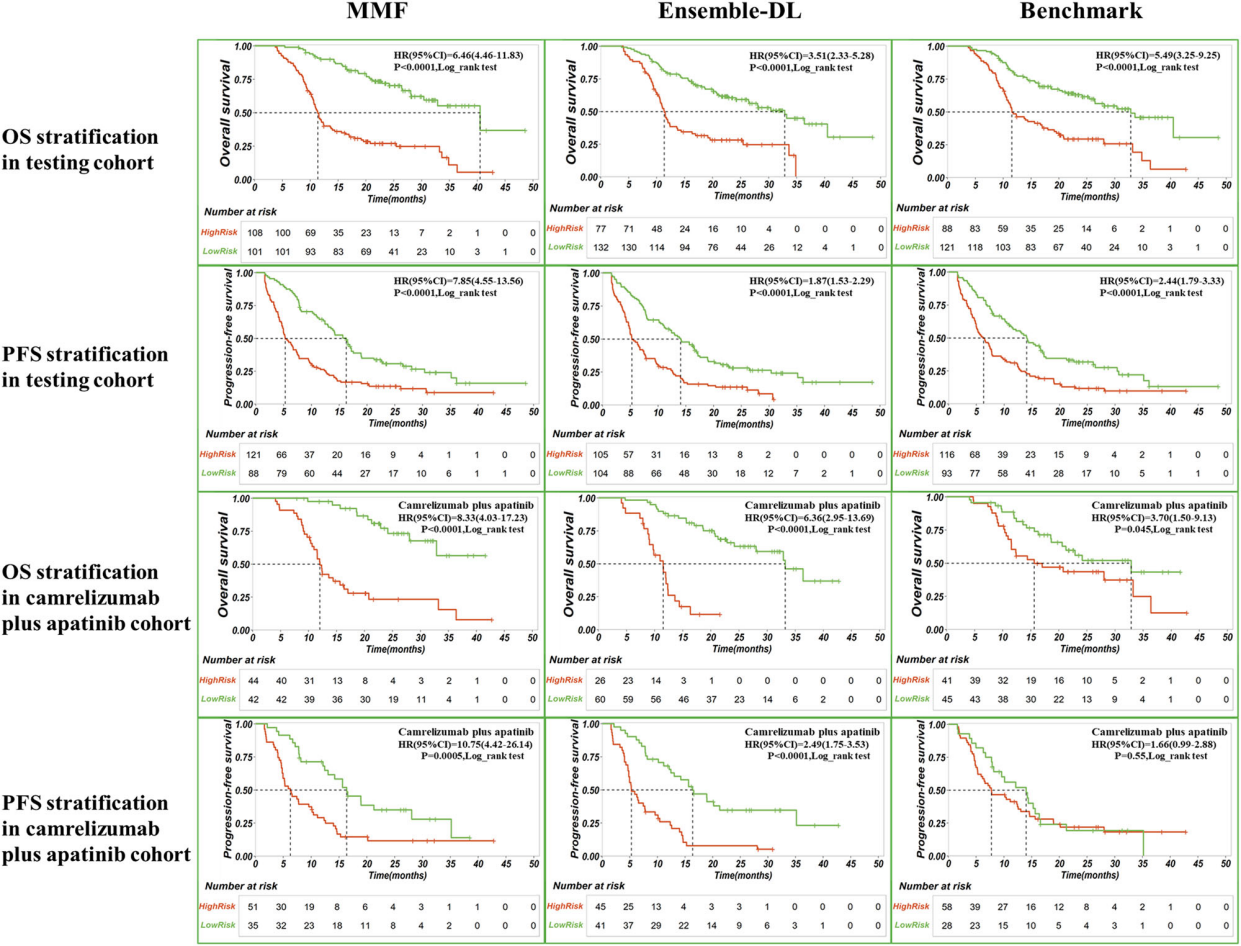
Predictive performance and risk stratification of the MMF system. The figure shows the predictive and risk stratification capabilities of the MMF, its core Ensemble-DL model, and the benchmark model for OS and PFS. The analysis includes an independent external testing cohort (encompassing all treatment regimens) and a cohort treated with camrelizumab plus apatinib. Kaplan-Meier curves illustrate the survival differences between high- and low-risk groups. Between-group differences were assessed by the log-rank test. Hazard ratios (HR) and their 95% confidence intervals (CI) quantify the risk increase in the high-risk group relative to the low-risk group. MMF multimodal fusion. Ensemble-DL ensemble deep learning, OS overall survival. PFS progression-free survival.

To evaluate the predictive performance of the MMF system, we compared it with seven established prognostic models. As shown in Tables 2, 3, the MMF system demonstrated significantly better performance in predicting OS and PFS than other models. Specifically, in the external test cohort, the mRECIST model had the lowest predictive performance, with a Harrell’s concordance index(C-index) of 0.57 (OS) and 0.58 (PFS), followed by the radiomics model, with a C-index of 0.58 (OS and PFS). In contrast, the MMF system demonstrated exceptional performance, achieving a C-index of 0.74 for OS and 0.69 for PFS, representing a 29.8% and 27.6% improvement in OS prediction over the mRECIST and radiomics models, respectively, as well as a 20.0% enhancement in PFS prediction compared to both models. The MMF system also outperformed the benchmark model (OS: 0.67, *p* = 0.0011; PFS: 0.65, *p* = 0.033) and the ensemble deep learning framework(Ensemble-DL) model (OS: 0.69, *p* = 0.0028; PFS: 0.66, *p* = 0.044). The results suggested that while the benchmark model based on clinical features and the Ensemble-DL model based on imaging features had prognostic utility, their performance remained inferior to that of the MMF system. By integrating multimodal data (including imaging features and clinical features), the MMF system achieved improved prediction accuracy and robustness. Furthermore, as shown in Table 4, the MMF system demonstrated superior time-dependent ROC performance, accurately predicting OS at 1, 2, and 3 years and PFS at 3, 6, and 12 months (Supplementary Table 1). Kaplan-Meier analyses further supported the risk stratification capabilities of the MMF system (Fig. 1). After categorizing patients into high- and low-risk groups based on risk scores from each model, the MMF system showed superior discrimination in terms of Δmedian OS and Δmedian PFS in the external testing cohort. Specifically, the MMF system achieved a Δmedian OS of 29.16 months, compared to 21.6 months for the Ensemble-DL model and 21.31 months for the benchmark model. Similarly, the MMF system’s Δmedian PFS was 11 months, outperforming the Ensemble-DL model (8.78 months) and the benchmark model (7.77 months).

**Table 2:** Comparative performance of the MMF system and seven other prognostic models for OS

Model	Training			Validation			Testing		
	C-index	HR	p*	C-index	HR	p*	C-index	HR	p*
mRECIST	0.55 (0.53,0.57)	0.46 (0.31,0.68)	<0.0001	0.55 (0.54,0.59)	0.46 (0.21,1.00)	<0.0001	0.57 (0.54,0.61)	0.41 (0.23,0.71)	<0.0001
Radiomic	0.73 (0.70,0.76)	7.69 (5.71,10.35)	<0.0001	0.62 (0.56,0.69)	2.91 (1.27,6.68)	0.0048	0.58 (0.53,0.64)	2.03(1.22,3.36)	<0.0001
Network 1	0.68 (0.65,0.72)	2.37 (2.00,2.81)	<0.0001	0.65 (0.59,0.72)	1.66 (1.21,2.27)	0.014	0.65 (0.60,0.70)	2.37 (1.68,3.35)	<0.0001
Network 2	0.74 (0.71,0.76)	1.64 (1.52,1.76)	<0.0001	0.65 (0.58,0.72)	1.25 (1.08,1.44)	0.0069	0.64 (0.59,0.69)	1.38 (1.21,1.56)	<0.0001
Network 3	0.72 (0.69,0.75)	1.25 (1.21,1.30)	<0.0001	0.68 (0.62,0.73)	1.24 (1.12,1.36)	0.013	0.68 (0.63,0.73)	1.23 (1.14,1.32)	0.0028
Benchmark	0.71 (0.68,0.74)	6.46 (4.62,9.04)	<0.0001	0.66 (0.59,0.73)	3.99 (2.12,7.52)	0.016	0.67 (0.62,0.72)	5.49 (3.25,9.25)	0.0011
Ensemble- DL	0.76 (0.73,0.79)	3.67 (3.13,4.30)	<0.0001	0.70 (0.64,0.76)	2.47 (1.67,3.66)	0.072	0.69 (0.64,0.73)	3.51 (2.33,5.28)	0.0028
**MMF**	**0.82 (0.79,0.84)**	**15.51 (11.66,20.63)**	–	**0.73 (0.68,0.79)**	**3.56 (2.06,6.1)**	–	**0.74 (0.70,0.78)**	**6.46 (4.46,11.83)**	-

Bold values indicate the best performance metrics for each comparison.

Data in parentheses are 95% CIs. *Indicates the comparison to the MMF system. The *p* values were calculated via two-sided z-test.

*Ensemble-DL* ensemble deep learning, *MMF* multimodal fusion, *OS* overall survival, *HR* hazard ratio.

**Table 3:** Comparative performance of the MMF system and seven other prognostic models for PFS

Model	Training			Validation			Testing		
	C-index	HR	p*	C-index	HR	*p* *	C-index	HR	p*
mRECIST	0.56 (0.55,0.58)	0.42 (0.31,0.58)	<0.0001	0.57 (0.53,0.60)	0.41 (0.21,0.79)	<0.0001	0.58 (0.55,0.61)	0.45 (0.30,0.68)	<0.0001
Radiomics	0.67 (0.65,0.70)	2.90 (2.4,3.48)	0.002	0.58 (0.52,0.63)	2.02 (1.11,3.68)	0.004	0.58 (0.54,0.63)	1.47 (1.10,1.95)	<0.0001
Network 1	0.62 (0.59,0.65)	2.04 (1.70,2.45)	<0.0001	0.63 (0.57,0.69)	1.87 (1.30,2.69)	<0.15	0.61 (0.57,0.65)	1.93 (1.39,2.67)	0.0002
Network 2	0.66 (0.64,0.69)	1.56 (1.43,1.70)	<0.0001	0.62 (0.56,0.67)	1.37 (1.13,1.67)	0.042	0.60 (0.55,0.64)	1.27 (1.11,1.46)	<0.0001
Network 3	0.65 (0.62,0.67)	1.26 (1.19,1.33)	<0.0001	0.61 (0.55,0.68)	1.20 (1.02,1.41)	0.012	0.64 (0.60,0.68)	1.54 (1.28,1.85)	0.012
Benchmark	0.66 (0.63,0.68)	2.71 (2.23,3.28)	<0.0001	0.64 (0.57,0.70)	2.31 (1.64,3.26)	0.14	0.65 (0.61,0.69)	2.44 (1.79,3.33)	0.033
Ensemble- DL	0.69 (0.66,0.71)	2.06 (1.82,2.33)	0.0006	0.64 (0.58,0.69)	1.63 (1.24,2.14)	0.051	0.66 (0.61,0.70)	1.87 (1.53,2.29)	0.044
**MMF**	**0.72 (0.69,0.74)**	**11.84 (8.38,16.72)**	-	**0.68 (0.62,0.74)**	**6.06 (3.06,11.98)**	-	**0.69 (0.65,0.73)**	**7.85 (4.55,13.56)**	-

Bold values indicate the best performance metrics for each comparison.

Data in parentheses are 95% CIs. *Indicates the comparison to the MMF system. The *p* values were calculated via two-sided z-test.

*Ensemble-DL* ensemble deep learning, *MMF* multimodal fusion, *PFS* progression-free survival, *HR* hazard ratio.

**Table 4:** Comparison of performance metrics between different prognostic models for predicting OS

Data Set and Model	1-year AUC(95%CI)	2-year AUC(95%CI)	3-year AUC(95%CI)
Discovery cohort(*n* = 650)			
mRECIST	0.57(0.54–0.59)^†^	0.56(0.52–0.60)^†^	0.62(0.53–0.79)*
Radiomic	0.77(0.73–0.81)^†^	0.74(0.69–0.78)^†^	0.60(0.49-0.70)^†^
Network 1	0.71(0.67–0.75)^†^	0.72(0.66–0.77)^†^	0.70(0.60-0.81)*
Network 2	0.77(0.73–0.81)^†^	0.78(0.73–0.83)*	0.75(0.65–0.85)
Network 3	0.77(0.73–0.81)^†^	0.75(0.70–0.81)^†^	0.64(0.52–0.76)^†^
Benchmark	0.76(0.72–0.80)^†^	0.75(0.66–0.84)^†^	0.75(0.66–0.84)
Ensemble DL	0.80(0.77–0.84)^†^	0.80(0.75–0.84)*	0.75(0.65–0.85)
**MMF**	**0.87(0.84–0.90)**	**0.84(0.80-0.88)**	**0.81(0.72–0.89)**
Testing cohort(*n* = 209)			
mRECIST	0.58(0.54–0.63)^†^	0.62(0.55–0.68)^†^	0.55(0.41–0.69)*
Radiomic	0.61(0.53–0.70)^†^	0.61(0.52–0.70)^†^	0.64(0.49–0.79)*
Network 1	0.72(0.65–0.79)*	0.67(0.59–0.76)*	0.80(0.67–0.93)
Network 2	0.71(0.64–0.78)*	0.71(0.62-0.79)*	**0.87(0.75–0.98)**
Network 3	0.76(0.69–0.83)	0.72(0.64–0.81)*	0.73(0.56–0.89)
Benchmark	0.70(0.63–0.78)^†^	0.72(0.64–0.80)*	0.66(0.50–0.83)*
Ensemble-DL	0.78(0.72–0.84)	0.74(0.66–0.82)*	0.83(0.72–0.94)
**MMF**	**0.81(0.75–0.87)**	**0.81(0.74–0.88)**	0.79(0.67–0.91)

Bold values indicate the best performance metrics for each comparison.

Values in parentheses represent 95% confidence intervals (CIs). AUC denotes the area under the receiver operating characteristic curve.

*P < 0.05 compared with the MMF system.

^†^*P* < 0.001 compared with the MMF system using the paired *t* test.

*Ensemble-DL* ensemble deep learning, *MMF* multimodal fusion, *OS* overall survival.

To validate the generalizability of the MMF system, we performed an additional subgroup analysis within both the discovery(training and validation) and external testing cohorts, specifically focusing on patients treated with camrelizumab and apatinib. This allowed us to assess the prognostic prediction and risk stratification capabilities of the MMF system in a clinically relevant setting. It is important to note that the training data for the MMF system included a variety of ICIs and targeted therapies, not just camrelizumab and apatinib. Despite the differing treatment regimens, the MMF system maintained excellent predictive performance, achieving a C-index of 0.78 (OS) and 0.71 (PFS) in the external testing cohort, outperforming all models except the Ensemble-DL model (all *p* < 0.0001) (Table 5). The MMF system also showed strong OS and PFS risk stratification abilities (log-rank test, all *p* < 0.0001) (Fig. 1). Additionally, the MMF system demonstrated consistent risk stratification across various clinical subgroups, including age, sex, tumor size/number, HBV status, Barcelona Clinic Liver Cancer(BCLC) stage, alpha-fetoprotein(AFP) levels, Eastern Cooperative Oncology Group(ECOG) status, portal vein tumor thrombosis(PVTT), metastasis pattern and prior treatment (Supplementary Fig. 1 and Supplementary Fig. 2). The robustness across subgroups highlights the potential of the MMF system to guide personalized treatment, enabling more tailored therapeutic decisions based on individual patient characteristics. Notably, for this specific treatment cohort, the Ensemble-DL model achieved predictive performance comparable to the MMF system. In the testing cohort, the Ensemble-DL model achieved a C-index of 0.75 (OS) and 0.70 (PFS), showing no significant difference from the MMF system (OS: *p* = 0.17; PFS: *p* = 0.71) but outperforming the benchmark model (OS: C-index = 0.64, *p* = 0.026; PFS: C-index = 0.59, *p* = 0.019). The Ensemble-DL model also showed robust performance in subgroup analyses (Supplementary Fig. 3 and Supplementary Fig. 4), reinforcing its role as the core of the MMF system. The success of the Ensemble-DL model was attributed to its ensemble strategy, which ensembled three complementary 3D neural networks and leveraged CT imaging features from the arterial, portal venous, and delayed phases. This strategy improved survival prediction performance compared to individual models, as reflected in a more stable C-index, higher hazard ratios, and better identification of low-risk patients (Tables 2, 3 and Supplementary Fig. 5). Additionally, to systematically evaluate the impact of different model architectures, we assessed multiple vision-based foundation models, including convolutional neural network (CNN)-based models(3D ResNet50^25^, 3D Desenet121^26^, 3D ConvNeXt^27^ and EfficientNet B1^28^), models utilizing different learning paradigms, and Transformer-based models(3D VIT^29^, 3D SwinUNETR^30^, 3D MaxVit^31^ and CNN-Transformer^32^). Experimental findings indicated that our final selected model combination—EfficientNet (CNN-based), the semi-supervised learning model, and the CNN-Transformer—consistently exhibited better C-index performance in both the validation and external testing cohorts (Supplementary Table 2). Ablation experiments further revealed that integrating features from all three CT imaging phases (arterial, portal venous, and delayed) and combining the three selected network architectures achieved the best network performance (Supplementary Tables 3, 4).

**Table 5:** Comparison of prognostic models for predicting OS and PFS in patients treated with camrelizumab plus apatinib

Model	Discovery (*n* = 324)^#^				Testing (*n* = 86)^#^			
	C-index	95%CI	*p*value^†^	*p*value*	C-index	95%CI	*p*value^†^	*p*value*
overall survival								
mRECIST	0.55	0.52,0.57	<0.0001	<0.0001	0.53	0.47,0.59	<0.0001	<0.0001
Radiomic	0.68	0.64,0.72	0.003	<0.0001	0.55	0.45,0.64	<0.0001	<0.0001
Benchmark	0.68	0.65,0.72	0.009	<0.0001	0.64	0.55,0.72	0.026	<0.0001
Ensemble-DL	0.74	0.71,0.78	–	<0.0001	0.75	0.68,0.81	–	0.17
MMF	0.79	0.76,0.82	<0.0001	–	0.78	0.72,0.84	0.17	–
progression-free survival								
mRECIST	0.55	0.53,0.57	<0.0001	<0.0001	0.56	0.51,0.60	<0.0001	<0.0001
Radiomic	0.66	0.63,0.69	0.075	0.0013	0.56	0.51,0.60	<0.0001	<0.0001
Benchmark	0.66	0.63,0.70	0.15	0.00025	0.59	0.52,0.66	0.019	<0.0001
Ensemble-DL	0.69	0.66,0.73	–	0.027	0.70	0.64,0.76	–	0.71
MMF	0.72	0.69,0.75	0.027	–	0.71	0.65,0.76	0.71	–

^†^ Indicates the comparison to the Ensemble DL model. * Indicates the comparison to the MMF system. ^#^ Denotes subgroup analyses within both the Discovery cohort (*n* = 324) and Testing cohort (*n* = 86), specifically referring to patients treated with camrelizumab plus apatinib. The *p* values were calculated via two-sided z-test.

*Ensemble-DL* ensemble deep learning, *MMF* multimodal fusion, *OS* overall survival, *PFS* progression-free survival.

### Interpretability and prognostic mechanism of MMF system

**Fig. 2:**
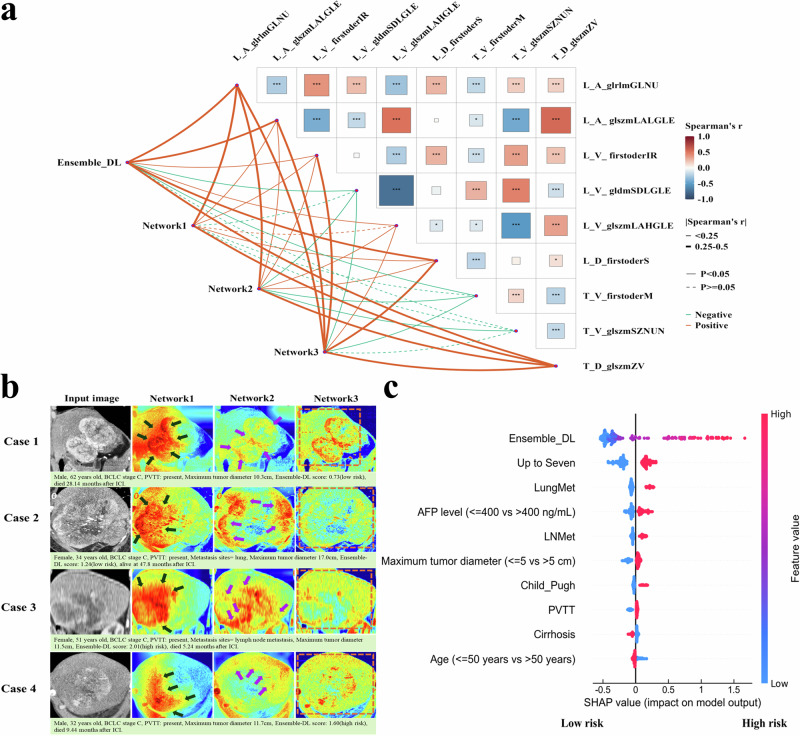
Correlation between ensemble-DL signature and radiomic features, model attention visualization, and SHAP analysis. **a** The Ensemble-DL signature and individual networks show varying degrees of correlation with 9 radiomics features extracted from multiphase CT scans. These features reflect tumor and liver heterogeneity as well as the complexity within the tumor, all of which are relevant to predicting overall survival (OS). The correlations suggest that the Ensemble-DL model captures important radiomic information related to tumor biology and prognosis. Line color indicates the direction of the correlation: red = positive, blue = negative. **b** Grad-CAM heatmaps visualize the regions contributing most to model predictions: Network 1 focuses on high-activity tumor areas (black arrows), highlighting proliferative or aggressive regions; Network 2 emphasizes the tumor boundary and surrounding microenvironment (purple arrows), capturing information related to tumor invasion; Network 3 captures global contextual features, including the entire liver and surrounding tissues (orange boxes), providing a broader anatomical perspective. **c** SHAP values illustrate the contributions of individual features to the MMF system’s survival predictions, where higher values (rightward shift) indicate increased risk (poorer prognosis) and lower values (leftward shift) correspond to lower risk (better prognosis). The color gradient represents feature values, with red denoting high values and blue indicating low values, providing additional insight into their impact on model predictions.

To gain a deeper understanding of the predictive mechanisms of the MMF system, we focused on analyzing the interpretability of its core component, the Ensemble-DL model, to uncover how it extracts key information from imaging data to enhance overall predictive performance. First, we analyzed the correlation between the Ensemble-DL signature and radiomics features to explore their biological significance. Radiomics features quantitatively described imaging data, encompassing complex characteristics of the tumor and its microenvironment, including texture, shape, and intensity distribution. We extracted 15 radiomics features associated with OS and PFS from arterial, portal venous, and delayed phases of contrast-enhanced CT images (listed in Supplementary Table 5). These features included gray-level histograms, gray-level run length matrix (GLRLM), gray-level size zone matrix (GLSZM), and first-order statistical features, capturing multidimensional information about the tumor and liver across different enhancement phases. Spearman correlation analysis (Fig. 2a) demonstrated a significant correlation between the Ensemble-DL signature and traditional radiomics features (r = 0.55, *p* < 0.0001), indicating high consistency. Specifically, features such as liver gray-level non-uniformity (L_A_glrlmGLNU) and low gray-level zone emphasis (L_V_glszmLAHGLE) from the arterial and portal venous phases were positively correlated with the Ensemble-DL signature, highlighting the importance of blood perfusion heterogeneity and tissue structure during these phases in prognostic prediction. In addition, tumor heterogeneity features (e.g., T_D_glszmZV) correlated positively with the Ensemble-DL signature, while first-order statistical features (e.g., T_V_firstorderM) showed a negative correlation, suggesting the complexity of internal tumor structure played a crucial role in prognosis. These results showed that the Ensemble-DL model effectively captured key information consistent with radiomics features and identified complex features related to prognosis by integrating data from multiple enhancement phases, thereby enhancing predictive accuracy. Similar findings were observed for PFS (Supplementary Fig. 6). This consistency also demonstrated the potential of radiomics in interpreting deep learning models and providing a transparent basis for quantifying tumor biology.

To further explore the predictive mechanism of the Ensemble-DL model, we used Gradient-weighted class activation mapping (Grad-CAM) (Fig. 2b) to analyze the critical image regions focused on by the individual networks during CT scan prediction. Activation maps reveal the importance of each pixel to the final prediction. Interestingly, while all networks focused on the tumor region, their specific areas of attention differed. Network 1 focused on active tumor components (black arrows). The activation map (Fig. 2b, second column) shows a strong response to highly active regions within the tumor, likely representing rapidly proliferating cells and other aggressive features, making Network 1 critical for capturing tumor activity relevant to prognosis. Network 2 focused on the tumor boundary and surrounding microenvironment (purple arrows). The activation map (Fig. 2b, third column) highlights the tumor edges and nearby tissues, allowing Network 2 to capture features related to tumor invasion and spread. Network 3 captured global image features (orange boxes). The activation map (Fig. 2b, fourth column) showed a response across the entire image, particularly the liver and surrounding tissues. This global analysis helped identify macro-level features, such as multiple liver lesions and spatial relationships to other organs, which were crucial for systemic assessment. To further enhance the interpretability of the MMF system’s predictions, we conducted a detailed Shapley Additive Explanations (SHAP) value analysis to assess the contribution of each feature. As shown in Fig. 2c, the Ensemble-DL signature had a greater impact on model predictions than clinical variables such as the Up-to-seven criteria, lung metastasis (LungMet), AFP level, and tumor size. Specifically, lower Shapley values for the Ensemble-DL signature were consistently associated with an increased predicted survival benefit, underscoring its critical role in the predictive model.

**Fig. 3:**
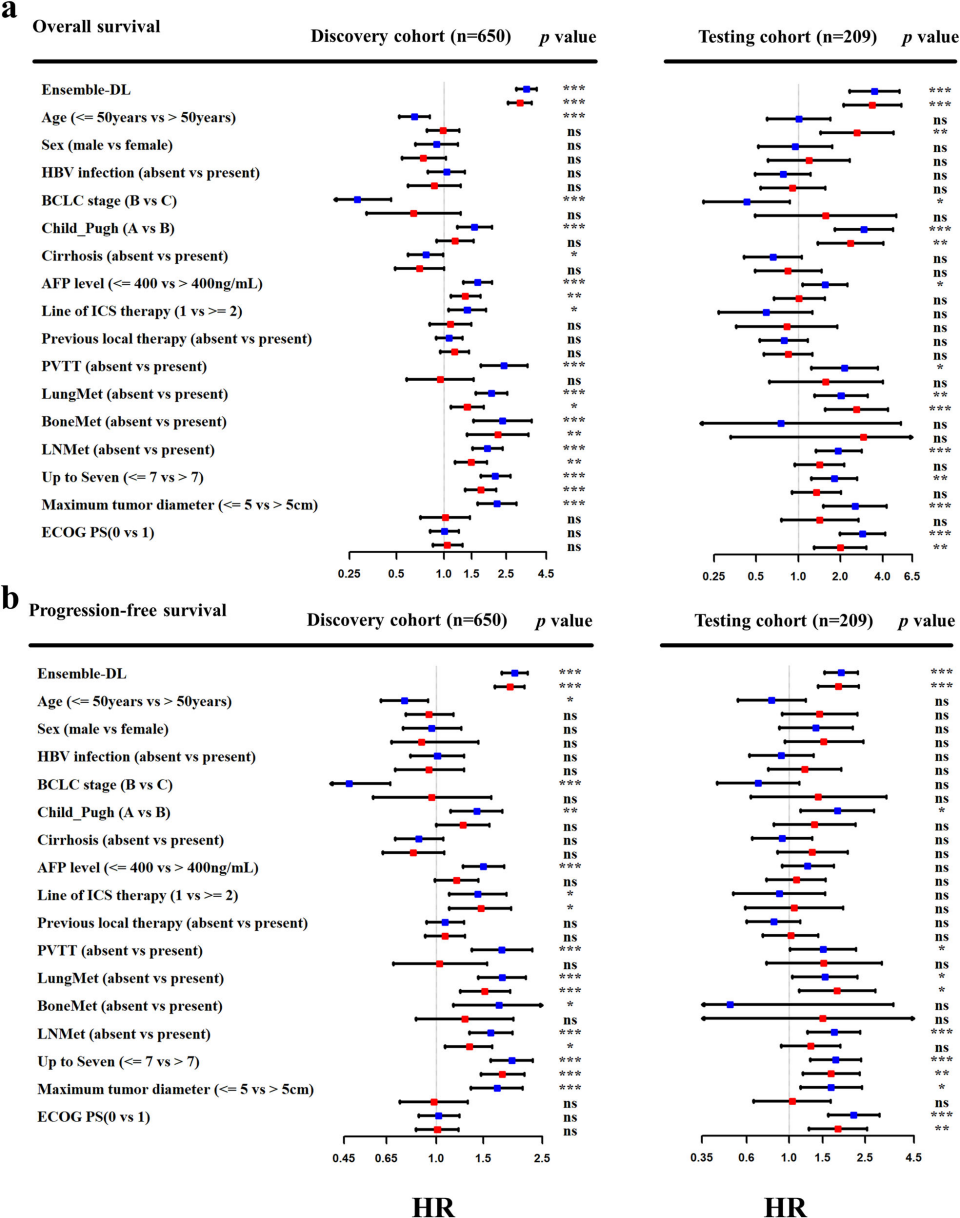
Incremental prognostic value of Ensemble-DL signature in clinical practice. The forest plot illustrates the hazard ratios (HR) and *p* values for the Ensemble-DL signature in predicting **a** overall survival and **b** progression-free survival. Blue bars represent univariate analysis, while red bars depict multivariate analysis. BCLC Barcelona Clinic Liver Cancer, AFP alpha-fetoprotein, PVTT portal vein tumor thrombus, LNMet lymph node metastasis, ECOG PS Eastern Cooperative Oncology Group Performance Status, ICIs immune checkpoint inhibitors. *p* values: *** <0.001, ** <0.005, * <0.05.

**Fig. 4:**
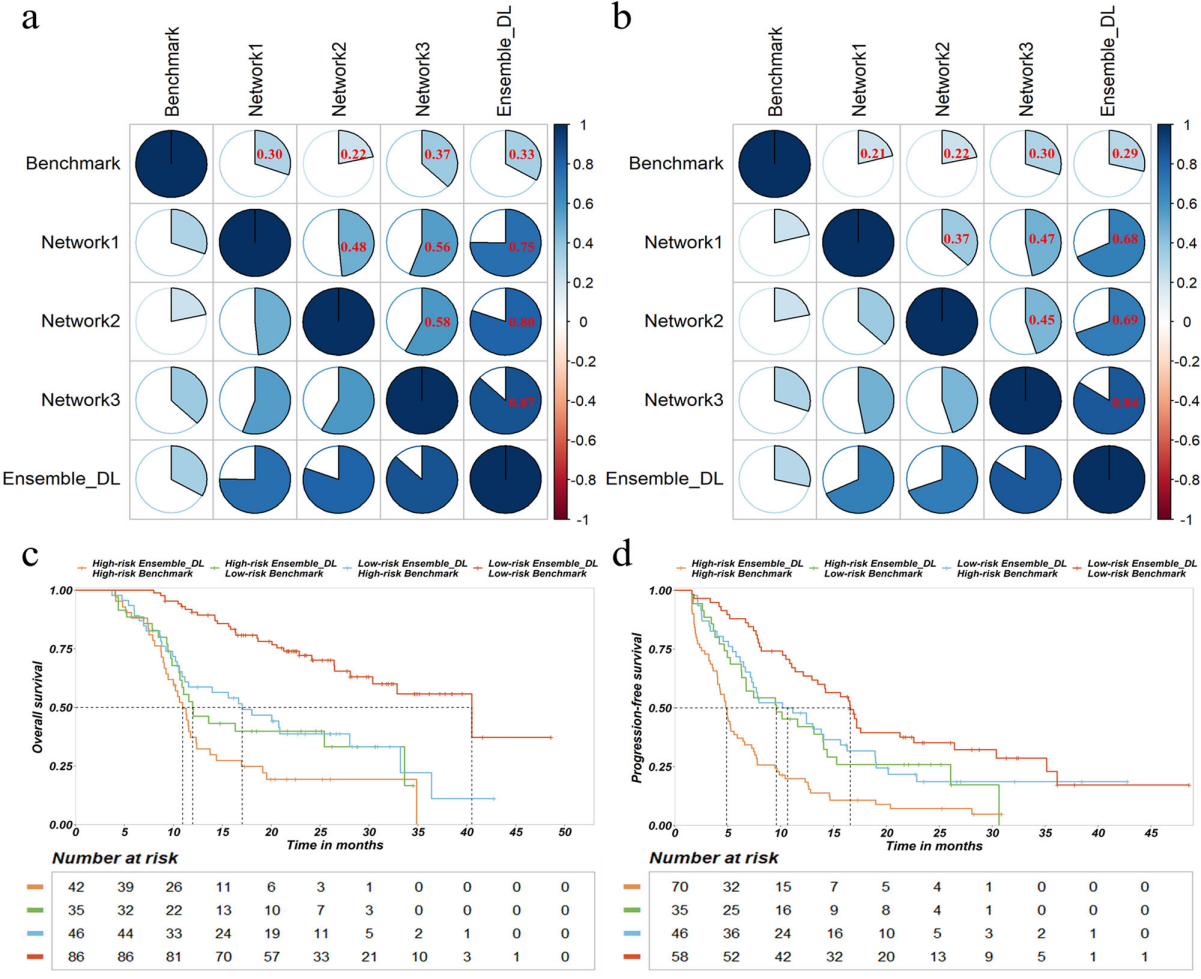
Enhanced prognostic value of ensemble-DL model on clinical benchmark. Correlation between deep learning signatures and clinical benchmark for (**a**) overall survival and (**b**) progression-free survival. Kaplan-Meier curves stratified by combined Ensemble-DL and benchmark model predictions on the testing cohort: (**c**) overall survival and (**d**) progression-Free Survival.

Finally, we compared the predictive ability of the Ensemble-DL signature with established clinical features. The Ensemble-DL signature showed significant OS associations in discovery and testing cohorts. While clinical factors such as liver function, AFP levels, PVTT, and metastasis patterns also showed associations, multivariate adjustment revealed that the Ensemble-DL signature remained the most critical predictor of OS (discovery cohort HR, 3.06 [95% CI: 2.4, 21.3]; *P* < 0.001; testing cohort HR, 3.38 [95% CI: 2.1, 5.4]; *P* < 0.001) (Fig. 3a). Similarly, univariate and multivariate PFS analyses revealed a consistent pattern (Fig. 3b). Detailed HR and p values are provided in the appendix (Supplementary Tables 6, 7). Correlation analysis showed a weak correlation between the Ensemble-DL signature and the benchmark model signature (r = 0.33, *p* < 0.0001 for OS; r = 0.29, *p* < 0.0001 for PFS, Fig. 4a, b), suggesting that the two models may capture different information relevant to the prognosis of HCC patients undergoing immunotherapy. Harmonized risk stratification by both models consistently identified low-risk and high-risk subgroups, correlating with the best outcomes and the worst prognosis, respectively (Fig. 4c, d). These findings indicated that the Ensemble-DL signature not only served as an independent prognostic factor for HCC patients but also effectively complemented existing clinical features. By integrating the strengths of the Ensemble-DL signature with clinical features, the MMF system further improved prediction accuracy and reliability, enabling more precise risk stratification and providing individualized treatment recommendations. These results demonstrated the significant potential of the MMF system for clinical application.

### Biologic significance of MMF system via ensemble-DL signature

**Fig. 5:**
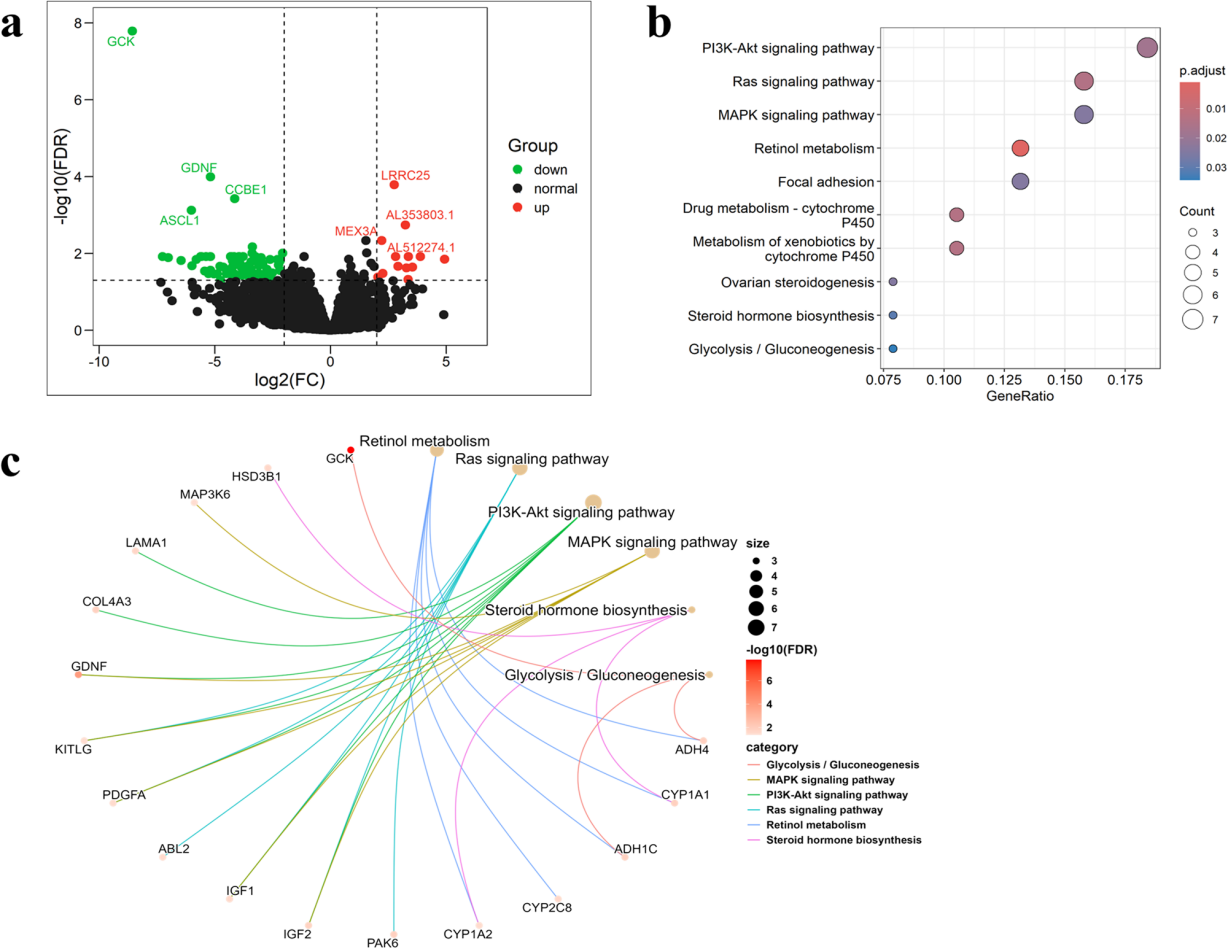
Differential gene expression. **a** Volcano Plot: Identifies differentially expressed genes (DEGs) associated with Ensemble-DL signature stratification. **b** KEGG enrichment analysis (bubble plot) shows functional enrichment of DEGs. **c** KEGG Centuple: Visualizes enriched pathways from the KEGG analysis of DEGs. DEGs differentially expressed gene, KEGG Kyoto Encyclopedia of Genes and Genomes.

To enhance the biological interpretability of the MMF system in predicting patient prognosis, we investigated the biological basis of the Ensemble-DL signature using the TCIA cohort. The Ensemble-DL model analyzed imaging features to classify patients into high- and low-risk groups based on an optimal cutoff value of 1.26, and this classification was strongly correlated with prognosis, improving the interpretability and reliability of the MMF system. Differential gene expression analysis identified 117 genes significantly associated with the Ensemble-DL stratification. Figure 5a illustrated the mutation frequencies of specific genes in stratified groups. For instance, the high-risk subgroups showed frequent mutations in LRRC25 and MEX3A, whereas the low-risk group exhibited frequent mutations in GCK, GDNF, CCBE1, and ASCL1, suggesting that these gene mutations might be linked to the risk stratification identified by the Ensemble-DL model and hinted at a potential connection between imaging features extracted by the model and underlying molecular mechanisms. Kyoto Encyclopedia of Genes and Genomes (KEGG) pathway enrichment analysis identified the PI3K-Akt signaling pathway, retinol metabolism, and MAPK signaling pathway as the main pathways associated with the differentially expressed genes(Fig. 5b, c). Analysis of the Ensemble-DL signature revealed imaging features linked to key biological pathways, enhancing the predictive power and interpretability of the MMF system for prognosis in HCC patients.

## Discussion

This study presents the MMF system aimed at accurately evaluating survival benefits in patients with unresectable HCC treated with ICIs. By integrating CT imaging-based deep learning features with clinical data through an innovative ensemble strategy, the MMF system overcomes the limitations of single-modality predictive models (e.g., tumor size, radiomics, or clinical data alone), enhancing the accuracy, robustness, and generalizability of survival predictions. The MMF system achieved C-index values of 0.74 and 0.69 for predicting OS and PFS in an external test set, respectively, outperforming comparative models. Moreover, the MMF system demonstrated good interpretability, with its core component, the Ensemble-DL signature, showing strong correlations with established radiomic features, as revealed by activation maps that effectively capture tumor characteristics from local, microenvironmental, and global perspectives, thereby aiding physicians in better understanding the prediction process and increasing trust in the outcomes. The MMF system also exhibited consistent risk stratification across various clinically relevant subgroups, confirming its wide clinical applicability.

Previous studies have attempted to use radiomics to predict the response of HCC patients to ICIs, but several limitations exist. For instance, some studies had small sample sizes or relied excessively on biomarkers such as PD-L1 expression, CD8 + , and CD3 + T-cell infiltration, which have limited predictive value in HCC^10,21,22,33^. Moreover, some studies used the mRECIST criteria to build short-term efficacy prediction models, neglecting OS, which is widely recognized as the gold standard for evaluating ICIs treatment in HCC. Both previous research^34^ and our findings suggest that mRECIST contributes minimally to OS prediction. Furthermore, research on deep learning models for predicting outcomes after ICIs treatment in HCC remains scarce. Xia et al.^18^ developed a prognostic model using baseline and follow-up CT images for survival prediction in HCC patients receiving immunotherapy, achieving a C-index of 0.70 and AUC of 0.70 and 0.65 for 1- and 2-year survival. The MMF system, using only pre-treatment CT and clinical data, outperformed with a C-index of 0.74 and AUCs of 0.87 and 0.84. These results demonstrate the MMF system’s superior accuracy, simplicity, and clinical practicality for individualized prognostic assessment in HCC immunotherapy.

Traditional radiomics approaches often rely on manual tumor segmentation and handcrafted feature extraction, which can be time-consuming and subjective^35,36^. This study utilizes a deep learning approach that automates feature extraction from the entire 3D liver region in triphasic CT images, overcoming these limitations and enhancing clinical usability. Ablation experiments demonstrated that neural networks combining triphasic CT information outperform single or dual-phase models, consistent with previous findings^11^. Previous deep learning studies have typically relied on single network architectures, which present clear limitations when addressing complex tasks like biomedical data analysis^37,38^. These limitations include overfitting, poor robustness, and distribution shifts. Additionally, the “black box” nature of deep learning models, with their lack of interpretability, hinders their clinical application^39^. To address these challenges, the MMF system employs an innovative ensemble learning strategy, integrating three distinct 3D neural networks for CT image feature analysis, which significantly enhances the model’s generalizability and robustness. While vision-based foundation models, particularly those employing Transformer architectures, have demonstrated significant advancements in robustness and generalization in recent years, current 3D Transformer models are primarily designed for image segmentation tasks. Consequently, their efficacy in classification and regression tasks remains limited. For example, 3D SwinUNETR^30^ achieved a C-index of only 0.57 for OS within the testing cohort. To address task-specific relevance and enhance clinical utility, this study implemented a three-model ensemble approach, comprising EfficientNet, a semi-supervised learning model, and a CNN-Transformer model. EfficientNet, a CNN architecture recognized for its computational efficiency, effectively extracts robust image features using limited parameters and computational resources. Its underlying design principles align with the objectives of vision-based foundation models and consistently exhibit superior performance compared to other CNN-based architectures. The semi-supervised learning model integrates autoencoder-based pre-training with DeepSurv fine-tuning techniques, synergistically combining the data efficiency of unsupervised learning with the task-specific precision of supervised learning. This integration enhances the model’s generalization capacity and reduces the tendency for overfitting. Furthermore, this study employs an optimized CNN-Transformer model based on the methodology proposed by Jang et al.^32^, effectively leveraging the inherent strengths of both 3D CNNs and Transformers. The CNN component efficiently extracts localized tumor features from CT images. Meanwhile, the Transformer component utilizes attention mechanisms across axial, coronal, and sagittal planes to model spatial relationships between tumor regions and surrounding liver parenchyma, capturing comprehensive spatial contextual information. Experimental results further demonstrated that the features extracted by the Ensemble-DL model were highly correlated with traditional radiomics features, such as those reflecting tumor and background liver parenchyma heterogeneity, and also outperformed traditional radiomics features in prediction tasks, with a 19.0% improvement in the C-index for OS prediction. This finding not only validates the effectiveness of deep learning models in feature extraction but also suggests that they can surpass traditional radiomics by capturing additional clinically relevant imaging information.

Additionally, Grad-CAM activation maps visually illustrated the advantages of the Ensemble-DL model, highlighting its ability to focus on different image regions using three networks and demonstrating its capability to capture complementary features from complex medical images. The synergistic operation of the three networks in the MMF system showcases the true value of ensemble learning: Network 1 analyzes tumor activity, Network 2 captures tumor boundaries and microenvironment, and Network 3 provides a global liver assessment, forming a comprehensive, multi-layered feature extraction framework. The results indicate that integrating different deep learning architectures allows for a more holistic understanding of tumors and surrounding tissues, thereby providing a more accurate and thorough prognostic assessment. Building on the Ensemble-DL signature, the MMF system further integrates key clinical characteristics of patients to construct a multidimensional information extraction framework, synthesizing prognostic information from both tumor and liver tissues. This integration not only enhances the prognostic prediction accuracy of the model but also effectively bridges the gap between deep learning models and traditional radiomics. The combination of the superior predictive performance of deep learning models and the interpretability of traditional radiomics features allows the MMF system to lead in both performance and explainability. SHAP analysis further decomposes the model’s decision-making process at an individual feature level, underscoring the critical role of the Ensemble-DL signature while elucidating the contributions of both deep learning-derived and clinical features to outcome prediction. This provides a visual representation of their integration within the MMF system, enhancing interpretability and facilitating a more transparent combination of deep learning and traditional clinical prognostic factors. By organically combining latent information from the “black box” model with Grad-CAM visualization, interpretable radiomic features, and SHAP analysis, the MMF system provides physicians with a tool that offers both precision and interpretability, significantly improving its acceptability and potential for clinical application.

Our study also benefited from large sample size and a high-quality dataset that included detailed clinical, laboratory, imaging, treatment regimen, and survival information, providing robust statistical support for model development and assessing potential confounding factors. The MMF system effectively captured a wide range of complex factors influencing patient survival outcomes by incorporating data from patients treated with various combinations of ICIs and molecularly targeted therapies. In an independent, multicenter external testing cohort, the MMF system demonstrated excellent performance in predicting OS and PFS. Notably, the Ensemble-DL model, the core component of the MMF system, achieved comparable predictive performance to the overall MMF system in a cohort of HCC patients receiving camrelizumab plus apatinib and outperformed the benchmark. Multivariate analysis revealed the Ensemble-DL signature to be an independent predictor of OS and PFS, demonstrating significantly better predictive efficacy compared to established clinical features, including liver function, AFP levels, PVTT, and metastasis patterns. This underscores its crucial role in prognosis prediction within the MMF system. In real-world clinical practice, the MMF system has the potential to provide physicians with more accurate prognostic assessments, thereby improving survival outcomes for HCC patients. By inputting the patient’s CT images and clinical indicators into the MMF system, physicians can obtain risk scores for OS and PFS after ICIs treatment. These risk scores, in conjunction with corresponding activation maps, can facilitate accurate prediction of patient prognosis and the development of personalized treatment strategies. The Ensemble-DL model can provide accurate and individualized prognostic assessments for HCC patients receiving camrelizumab plus apatinib combination therapy. For patients receiving other ICIs and molecularly targeted therapy combinations, the MMF system, which integrates Ensemble-DL signature and clinical features, would be a more suitable choice. To our knowledge, this multicenter study pioneers using an imaging-based ensemble deep learning model to predict survival benefits from ICIs in unresectable HCC.

Gene expression analysis identified GCK and LRRC25 as pivotal genes in stratifying low and high-risk groups. GCK overexpression inhibits HCC proliferation via lactate accumulation and energy crisis induction, suggesting it functions as a tumor suppressor gene^40^. Conversely, LRRC25, suppressing RIG-I, aids tumor immune evasion, highlighting it as a potential target for developing new HCC immunotherapy strategies^41^. Functional network analysis highlighted the PI3K/Akt pathway enrichment among differentially expressed genes. Activating this pathway in HCC reduces immunotherapy sensitivity via PD-L1 and VEGF upregulation^42^. By integrating imaging phenotypes with molecular profiles, this study revealed a strong association between risk stratification based on the Ensemble-DL signature and the activity of GCK, LRRC25, and the PI3K/Akt signaling pathway. Although this study primarily focused on HCC, the findings have broader implications for other malignancies. LRRC25-mediated immune evasion may play a critical role in RIG-I-dependent cancers(e.g., melanoma, colorectal cancer, and non-small cell lung cancer), suggesting that combining LRRC25 inhibition with ICIs could enhance therapeutic outcomes in these contexts^43,44^. Additionally, the metabolic vulnerability associated with GCK aligns with the classical Warburg effect^45^. Modulating GCK activity to disrupt tumor cell energy metabolism and induce an “energy crisis” may represent a promising strategy for targeting metabolically active solid tumors. Furthermore, the study highlights the clinical potential of PI3K/Akt inhibitors. These agents have demonstrated significant progress in breast cancer treatment, with several inhibitors (e.g., alpelisib and capivasertib) already approved and are currently under active investigation in other malignancies(e.g., ISRCTN16426935 and PNOC001)^46–48^. Our findings support the initiation of clinical trials in HCC patients, suggesting that, for the high-risk HCC subgroup characterized by activated PI3K/Akt signaling, combining PI3K/Akt inhibitors with ICIs may offer a more effective therapeutic strategy.

Despite these promising findings, the study has limitations. Firstly, as a retrospective, real-world study, it was subject to potential biases and confounding factors. While the model performed well in the independent testing cohort, further validation in larger, prospective studies is necessary. Secondly, while the deep learning signature aided in selecting patients for current standard treatments (ICIs combined with molecular targeted therapy), emerging immunotherapies necessitate further testing in different cohorts. Thirdly, enhancing the interpretability of deep learning models is crucial. Although we observed correlations between the deep learning signature, handcrafted radiomic features, gene mutations, and signaling pathways, these associations were preliminary. In future research, we plan to conduct prospective multicenter trials involving a broader range of HCC patients with diverse clinical and molecular characteristics. These studies will specifically examine various immunotherapy regimens, including dual checkpoint blockade (PD-1/PD-L1 + CTLA-4 inhibition) and personalized neoantigen-based vaccines. Through these trials, we aim to validate our ensemble deep learning model further and assess whether deep learning-derived imaging features can predict treatment responses across different immune-targeting mechanisms, thereby clarifying its clinical utility for personalized treatment decisions. Additionally, ongoing exploration and integration of advanced vision-based foundation models into our analytical pipeline will enable dynamic model updates, facilitating adaptation to evolving clinical practices and potentially enhancing generalizability and predictive performance. Furthermore, validation of differentially expressed genes and key pathways in a larger cohort aims to elucidate the biological basis of radiogenomic associations, establish causal relationships, and further explore the potential of multimodal AI to advance precision oncology, including risk stratification and biomarker-driven clinical trials, while enhancing the interpretability of deep learning predictions.

In conclusion, this study developed and validated the MMF system, which integrates CT-based deep learning with clinical features to assess survival benefits in unresectable HCC patients receiving ICIs therapy. By leveraging ensemble learning, the system enhanced prediction accuracy and robustness. Its predictive power and interpretability offer personalized guidance for physicians. Future research will focus on prospective clinical studies to further validate and optimize the MMF system in real-world settings, positioning it as a key tool for decision-making in HCC immunotherapy and advancing personalized precision medicine to improve patient outcomes.

## Methods

### Study design and participants

**Fig. 6:**
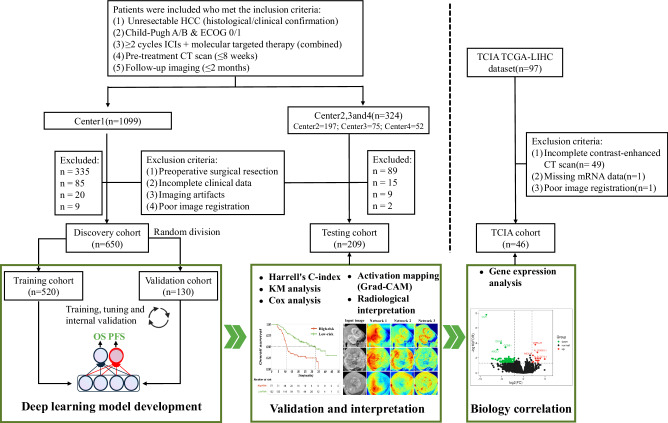
Patient selection flowchart and study design. ECOG Eastern Cooperative Oncology Group (ECOG) performance, TCGA-LIHC The Cancer Genome Atlas Liver Hepatocellular Carcinoma, TCIA the Cancer Imaging Archive, HCC hepatocellular carcinoma, ICIs immune checkpoint inhibitors, Grad-CAM Gradient-weighted activation mapping method, KM Kaplan-Meier curves.

This retrospective multicenter study included 859 patients with unresectable HCC who received ICIs treatment at four independent institutions between July 1, 2019, and July 31, 2023. The study design is illustrated in Fig. 6. Inclusion criteria: (a) Histological or clinical confirmation of unresectable HCC. (b) Child-Pugh A/B liver function, ECOG performance status 0/1. (c) Combined ICIs and molecular targeted therapy for ≥2 cycles. (d) Underwent contrast-enhanced CT within 8 weeks before ICIs treatment and follow-up CT within 2 months for mRECIST assessment. Exclusion criteria: (a) History of HCC surgical resection. (b) Incomplete clinical or follow-up information. (c) Imaging artifacts or poor image registration quality. This retrospective multicenter study complied with the Helsinki Declaration and was approved by the Ethics Committees of the First Affiliated Hospital of the University of Science and Technology of China, the First Affiliated Hospital of Wannan Medical College, the First Affiliated Hospital of Anhui Medical University, and the Hefei Cancer Hospital of the Chinese Academy of Sciences (Approval Nos.: 2023-ky197, 2025-73, PJ 2025-03-61, SL-KY2024-045, respectively). Due to the retrospective design, informed consent was waived.

The discovery cohort was sourced from the First Affiliated Hospital of the University of Science and Technology of China(Center 1) and randomly divided (8:2) into training and validation cohorts for model training, tuning, and internal validation. The external testing cohort used data from the First Affiliated Hospital of Anhui Medical University(Center 2), the First Affiliated Hospital of Wannan Medical College(Center 3), and the Hefei Cancer Hospital of the Chinese Academy of Sciences(Center 4). Additionally, the study included 46 patients from the TCIA with complete contrast-enhanced CT and RNA sequencing information for gene expression analysis.

### CT study protocols, treatment protocol, follow-up, and mRECIST assessments

We retrospectively evaluated abdominal contrast-enhanced CT scans in patients receiving diverse ICIs and targeted therapies following established Chinese guidelines. Two experienced radiologists (J.L. and Z.Z., each with over eight years of experience) employed mRECIST criteria to assess tumor response after the initial CT and on subsequent scans performed every 6–9 weeks, with additional assessments quarterly until the study conclusion on March 31, 2024, or patient death. The coprimary endpoint was OS, and the secondary outcome measure was PFS. OS was calculated from the initiation of ICIs treatment to death from any cause. PFS was measured from the initiation of ICIs to disease progression per mRECIST criteria or death. Detailed protocols and summaries of specific ICIs and other treatment regimens are provided in Supplementary Information 1 and Supplementary Table 8.

### Image preprocessing

We implemented a standardized image preprocessing workflow to address the heterogeneity in CT data resulting from diverse CT scanners and imaging protocols and improve the generalizability of our deep learning models. Three-phase CT images, including arterial, portal venous, and delayed phases, were obtained from the Picture Archiving and Communication System (PACS). To segment the liver in each phase, we utilized a pre-trained nnUNet model, which automatically segment the liver parenchymal regions^49^. Subsequently, we employed Elastix software^50^ (version 5.0.1, https://github.com/SuperElastix/ElastixModelZoo) with the registration parameter pack (Par0057Bspline) to perform image registration, using the portal venous phase liver as the reference. Following the registration process, we resampled the registered images to a standardized 1 × 1 × 1 mm voxel size using cubic interpolation, while the masks were interpolated using nearest neighbor. To further enhance image quality and reduce noise, we applied an adaptive histogram equalization (CLAHE) filter^51^. This filter improved the contrast of the images and reduced the impact of noise, which can adversely affect the performance of deep learning models. Additionally, we set the Hounsfield Unit (HU) range to [-200, 250] to exclude irrelevant information and focus on the region of interest. To ensure comprehensive coverage of both liver parenchyma and tumor lesions, we used the 3D bounding box of the segmented liver mask to crop the entire liver region from the CT images. Finally, the harmonized 3D CT images were cropped to a size of 128 × 128 × 128 to match the model’s input and stacked into a 4D matrix for deep learning model training.

### Model development

**Fig. 7:**
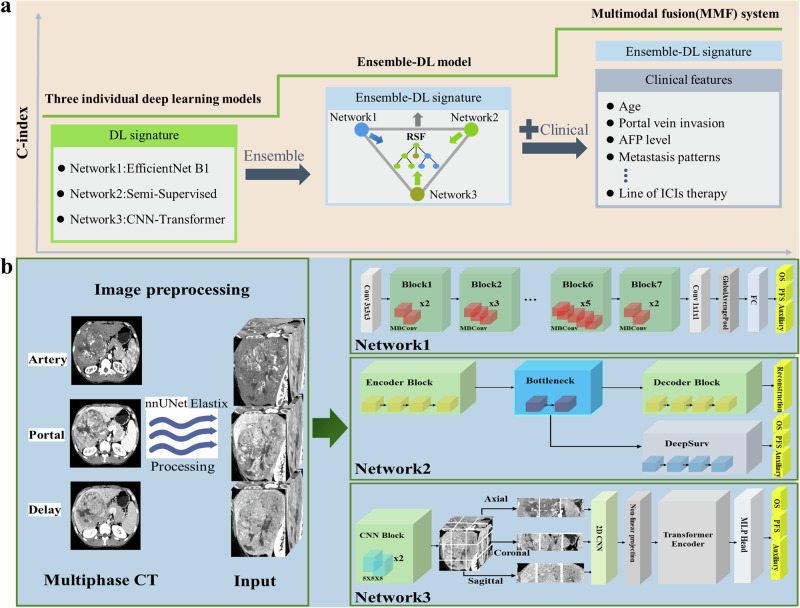
Construction and architecture of the MMF system. **a** The MMF system is developed stepwise, starting with individual deep learning (DL) models, progressing to the Ensemble-DL model, and finally forming the complete MMF system. The C-index increases with each iteration, indicating improved predictive performance. The core of the MMF system, the Ensemble-DL model, integrates three 3D neural networks to extract imaging features, which are then combined with clinical features using RSF. **b** The Ensemble-DL model ensembles three 3D neural networks (Network 1, Network 2, Network 3) that work together to extract imaging features from preprocessed multiphase CT images (arterial, portal venous, and delayed phases). Image preprocessing is performed using nnUNet and Elastix to generate input for the networks and to ensure data quality and consistency. MMF, multimodal fusion. Ensemble-DL, ensemble deep learning. RSF, random survival forest. MBConv, mobile inverted bottleneck convolution.

The workflow for constructing the MMF system, illustrated in Fig. 7a, showed a stepwise approach from individual network models to the Ensemble-DL model and, ultimately, to the complete MMF system. The core component of the MMF was the Ensemble-DL model, which integrated three complementary 3D neural networks to extract imaging features related to prognosis from multiple dimensions. The structure and feature extraction process of each network are illustrated in Fig. 7b and Supplementary Information 2. Network 1 was a CNN architecture based on EfficientNet B1^28^, which focused on extracting local information from within the tumor tissue to extract key features related to tumor characteristics at a microscopic level^52^. Network 2 was a semi-supervised hybrid model that integrated peritumoral tissue to capture features of the tumor microenvironment. This architecture included an encoder, bottleneck module, and decoder, which collectively captured spatial information at multiple scales, combined with a DeepSurv^53^ module for prognostic analysis, revealing the influence of the tumor microenvironment on patient survival. Network 3 was a CNN-Transformer architecture^32^ to capture global contextual information, including tumor heterogeneity, spatial distribution, and relationships with adjacent structures^54^. All three networks used preprocessed 3D CT images as input to ensure data quality and consistency. A multitask learning framework was employed, with different output channels predicting risk scores for OS and PFS, quantifying the risks of death and disease progression, respectively higher scores indicated greater risk. The risk scores from each network were combined using a Random Survival Forest (RSF)^55^ to form the Ensemble-DL signature. The primary advantage of the Ensemble-DL model lies in its ability to enhance overall performance by integrating the predictions of multiple models, thereby reducing biases and improving robustness and generalizability. This ensemble approach demonstrated superior effectiveness in recent deep learning applications, particularly in medical image analysis^56^. Building on this, the RSF was employed further to integrate the Ensemble-DL signature with key clinical features, leveraging their complementary strengths to develop the final MMF system for multimodal prognosis evaluation. The clinical features included key variables such as demographics (age, sex), clinical and laboratory markers (AFP levels, HBV infection, liver function, cirrhosis status, ECOG performance status), tumor characteristics (BCLC stage, tumor burden based on the Up-to-seven criteria^57,58^, metastasis pattern, PVTT, tumor size and number), and treatment history (treatment lines, prior local therapy). To ensure the model’s reliability and clinical applicability, we first trained it using a training cohort and then internally validated it with a validation cohort. External validation was subsequently performed on an independent testing cohort to confirm its clinical utility in survival prediction. This systematic development and validation process demonstrates the robustness and accuracy of the MMF system in survival prediction, providing a reliable basis for clinical decision-making. Additionally, to comprehensively assess the clinical incremental value of the MMF system and explore the crucial role of the Ensemble-DL signature in multimodal integration, the following comparative models were also constructed:

#### mRECIST model

A prediction model built using Cox proportional hazards regression^55^ based on mRECIST assessment results from the first follow-up CT, used to evaluate the incremental value of the MMF system compared to traditional tumor size-based methods.

#### Benchmark model

A prediction model built on existing clinical risk factors to assess the incremental value and complementarity of Ensemble-DL signature relative to clinical risk factors. Initially, the prognostic significance of individual features was assessed using univariate Cox proportional hazards models. To further explore interactions between different features, we used a multivariable analysis based on RSF with three-fold cross-validation. The model with optimal performance in the discovery cohort was selected and subsequently validated in external testing cohort to confirm its robustness and generalizability.

#### Radiomics model

A radiomics prognostic model constructed using RSF with radiomics features extracted from tumor and liver regions using a semi-automatic segmentation method, used for comparison and to enhance the interpretability of the Ensemble-DL model. Radiomic features were extracted using Python and the PyRadiomics library^59^. These features were derived from three-dimensional tumor and liver regions and included: shape features (*n* = 14), intensity features (*n* = 18), and texture features (*n* = 75). All feature definitions adhered to the Imaging Biomarker Standardization Initiative (IBSI) standards^60^. In total, 214 interpretable radiomic features were extracted from each 3D CT scan, resulting in 642 features from the three-phase contrast-enhanced CT images for each patient. To ensure robust radiomic feature extraction, a semi-automatic segmentation method was employed: The nnUNet model automatically segmented the images. Automated liver and tumor segmentation was performed and subsequently refined by an expert abdominal radiologist (J.X.) with eight years of experience. Poorly segmented masks underwent manual correction to optimize accuracy and quality. Images were standardized to a 1 × 1 × 1 mm voxel size using B-spline interpolation and normalized by discretizing voxel intensity values into 25 HU bins. A three-step procedure was developed within the training cohort to extract robust radiomic features.

(1) Univariate Analysis: univariate Cox proportional hazards models were employed to assess the prognostic significance of individual radiomic features in HCC patients. Each radiomic feature was used to construct a survival analysis model, with the prognostic power evaluated using the C-index. The feature selection process is expressed as:

1 $${F}_{{uni}}\,=\,\left\{f|{C}_{index}\left(CPH\left(f,\,y\right)\right) > {t}_{uni},\,f\in F\right\}$$

where *F* represents the complete set of extracted radiomic features, *F*_*uni*_ denotes the subset of features selected through univariate analysis and *f* is an individual radiomic feature. *y* represents the survival outcome (OS or PFS), while ${CPH}\left(f,{y}\right)$ refers to the Cox proportional hazards model applied to feature *f* and survival outcome y. The C-index, denoted as ${C}_{{index}}\left({CPH}\left(f,{y}\right)\right)$ quantifies the predictive strength of *f*, and *t*_*uni*_ is the univariate analysis threshold (set at 0.55).

(2) Feature Redundancy Reduction: used the Variance Inflation Factor (VIF)^61^ to eliminate unnecessary duplicate features. The process is expressed as follows:

2 $${F}_{{vi}f}={F}_{{uni}}-\left\{f|VIF(f,{F}_{uni}-f) > {t}_{vif},\,f\in {F}_{uni}\right\}$$

where *F*_*uni*_ represents the subset of features selected from univariate analysis, and *F*_*vif*_ denotes the refined subset of features retained after eliminating redundant ones. *f* is an individual radiomic feature, while ${VIF}(f,\,{F}_{{uni}}-f)$ measures the multicollinearity of feature *f* concerning the remaining features in *F*_*uni*_. $t$_*vif*_ is the threshold for multicollinearity (set at 10).

(3) Multivariable Analysis: Performed using a Random Survival Forest (RSF) to explore interactions between different features. The process is expressed as follows:

3 $${F}_{{mul}}=\left\{f|Score\left(RSF(f,{F}_{vif},y)\right) > {t}_{mul},f\in {F}_{vif}\right\}$$

where *F*_*vif*_ represents the subset of features retained after multicollinearity filtering, and *F*_*mul*_ denotes the final subset of selected features used for model training. *f* is an individual radiomic feature, while ${Score}({RSF}(f,\,{F}_{{vi}f},{y}))$ represents the importance score assigned to feature *f* by the RSF model based on its contribution to predicting survival outcome *y*. $t$_*mul*_ is the threshold for multivariable analysis (set at 0.05). Based on the selected radiomic features, RSF models were constructed to predict PFS and OS separately. The model was built on the discovery cohort and locked for validation.

### Statistical analysis

Statistical analyses were performed using Python 3.7.3 and R 4.3.0 by J.X. and T.W. Model performance was assessed by C-index, time-dependent ROC curves, and Cox proportional hazards models with HR and 95% confidence intervals (CI). Kaplan-Meier curves visualized survival by risk strata, and log-rank tests assessed differences between curves. Log-rank tests in training and validation cohorts determined optimal risk score cutoffs. For the MMF system and the Ensemble-DL model, individuals with OS and PFS risk scores ≥1.26, ≥1.98 and ≥0.45, ≥0.90, respectively, were classified into the high-risk group, while those with scores below these thresholds were categorized as low-risk. Grad-CAM was used to visualize image regions critical for model predictions. SHAP^62^ computed Shapley values, quantifying the contribution of specific features within the MMF system to individual predictions. Spearman’s rank correlation was used to analyze the correlation between the Ensemble-DL signature and radiomics features and clinical characteristics. *x*^2^ or Fisher’s exact tests were used to compare categorical variables, and *t* tests were used to compare continuous variables. Statistical significance was defined as *p* < 0.05 (two-tailed).

## Supplementary information

Supplementary information

## Data Availability

The datasets used and/or analyzed during the current study are available from the corresponding author upon reasonable request.

## Code availability

The code for deep learning models and biological function exploration is available at https://github.com/wtf633/Ensemble-DL-for-Predicting-Survival-Benefits.
