# Supplementary material for: A multimodal fusion system predicting survival benefits of immune checkpoint inhibitors in unresectable hepatocellular carcinoma
Source: NPJ Precis Oncol. 2025 Jun 14;9:185. doi: 10.1038/s41698-025-00979-6 (PMC12167364; doi:10.1038/s41698-025-00979-6)
Supplement: Supplementary file 1 — Supplementary information [file 41698_2025_979_MOESM1_ESM.pdf]

SUPPLEMENTARY INFORMATION 2

1. Supplementary Tables ***2***

Supplementary Table 1. Comparison of performance metrics between different prognostic models for predicting PFS. 2

Supplementary Table 2. Performance comparison of deep learning models for HCC prognosis prediction. 3

Supplementary Table 3. Ablation analysis to optimize Network 1 to Network 3 in overall survival and progression-free survival. 4

Supplementary Table 4. Ablation study evaluating different combinations of individual network models. 5

Supplementary Table 5. Radiomic features selected in the pre-treatment CT images on overall survival and progression-free survival. 6

Supplementary Table 6. Univariable and multivariable analyses of variables for overall survival in the discovery cohort and testing cohort. 7

Supplementary Table 7. Univariable and multivariable analyses of variables for Progression-free survival in the discovery cohort and testing cohort. 8

Supplementary Table 8. Agents administration protocol 9

2. Supplementary Figures ***10***

Supplementary Fig. 1. Subgroup analyses of MMF system for overall survival in testing cohort. 10

Supplementary Fig. 2. Subgroup analyses of MMF system for progression-free survival in testing cohort. 11

Supplementary Fig. 3. Subgroup analyses of Ensemble-DL model for overall survival in testing cohort. 12

Supplementary Fig. 4. Subgroup analyses of Ensemble-DL model for progression-free survival in testing cohort. 13

Supplementary Fig. 5. Stratified Kaplan-Meier curves for OS and PFS in the testing cohorts with Ensemble-DL and individual deep learning models. 14

Supplementary Fig. 6. Deep Learning-Radiomics Correlation: Associations between deep learning signatures and radiomics features related to progression-free survival. 15

3. Supplementary Information 1: CT imaging Protocol, Administration of ICIs and Assessments ***16***

CT Imaging Protocol 16

Administration of Immune Checkpoint Inhibitors and Assessments 16

4. Supplementary Information 2: Structure of three deep learning networks ***17***

5. Supplementary References ***19***

Supplementary Information

1. Supplementary Tables

Supplementary Table 1. Comparison of performance metrics between different prognostic models for predicting PFS.

Data Set and Model	3-month AUC(95%CI)	6-month (95%CI)	12- month AUC(95%CI)
**Discovery cohort(n= 650)**			
mRECIST	0.60(0.58-0.61) †	0.60(0.57-0.62) †	0.60(0.56-0.63) †
Radiomic	0.73(0.68-0.77)	0.73(0.69-0.76) *	0.69(0.64-0.73) †
Network 1	0.70(0.65-0.75) *	0.67(0.63-0.71) †	0.67(0.62-0.71) †
Network 2	0.72(0.68-0.77)	0.73(0.69-0.77) †	0.71(0.67-0.75) †
Network 3	0.70(0.66-0.75) *	0.68(0.64-0.72) †	0.69(0.64-0.73) †
Benchmark	0.68(0.63-0.74) †	0.71(0.67-0.75) †	0.70(0.66-0.74) †
Ensemble-DL	0.75(0.74-0.79)	0.75(0.71-0.78) †	0.73(0.69-0.78) *
MMF	0.77(0.72-0.81)	0.79(0.76-0.82)	0.78(0.74-0.81)
**Testing cohort(n= 209)**			
mRECIST	0.62(0.59-0.65) *	0.60(0.56-0.65) †	0.62(0.56-0.67) *
Radiomic	0.63(0.52-0.74)	0.62(0.54-0.70) †	0.64(0.56-0.72) *
Network 1	0.62(0.52-0.72) *	0.68(0.61-0.75) †	0.66(0.59-0.74) *
Network 2	0.62(0.52-0.72) *	0.67(0.60-0.75) †	0.65(0.58-0.73) *
Network 3	0.66(0.56-0.77)	0.75(0.69-0.82)	0.70(0.62-0.77) *
Benchmark	0.69(0.60-0.78)	0.75(0.68-0.81) *	0.70(0.63-0.77)
Ensemble-DL	0.67(0.57-0.77)	0.76(0.69-0.82) *	0.73(0.66-0.80)
MMF	0.72(0.63-0.81)	0.81(0.74-0.87)	0.76(0.69-0.82)

Note.—Values in parentheses represent 95% confidence intervals (CIs). AUC denotes the area under the receiver operating characteristic curve. Ensemble-DL, ensemble deep learning. MMF, multimodal fusion. PFS, progression-free survival.

* Represents $P < .05$ compared with the MMF system.

† Represents $P < .001$ compared with the MMF system using the paired t test.

5

Supplementary Table 2. Performance comparison of deep learning models for HCC prognosis prediction.

Models	OS			PFS		
	Training	Validation	Testing	Training	Validation	Testing
Network 1: CNN-based models						
3D ResNet50	0.64(0.61,0.68)	0.64(0.57,0.71)	0.62(0.57,0.67)	0.60(0.57,0.63)	0.62(0.56,0.68)	0.60(0.55,0.65)
3D Desenet121	0.65(0.61,0.69)	0.65(0.59,0.71)	0.61(0.55,0.66)	0.61(0.58,0.64)	0.59(0.47,0.65)	0.60(0.56,0.64)
3D ConvNeXt	0.69(0.65,0.72)	0.61(0.43,0.66)	0.59(0.54,0.65)	0.64(0.62,0.67)	0.56(0.48,0.63)	0.56(0.50,0.61)
**EfficientNet B1(ours)**	**0.68(0.65,0.72)**	**0.65(0.59,0.72)**	**0.65(0.60,0.70)**	**0.62(0.59,0.65)**	**0.63(0.57,0.69)**	**0.61(0.57,0.65)**
Network 2: Different learning paradigms						
Unsupervised	0.59(0.53,0.65)	0.54(0.41,0.66)	0.54(0.45,0.64)	0.58(0.52,0.63)	0.52(0.42,0.63)	0.53(0.45,0.61)
**Semi-supervised(ours)**	**0.74(0.71,0.76)**	**0.65(0.58,0.72)**	**0.64(0.59,0.69)**	**0.66(0.64,0.69)**	**0.62(0.56,0.67)**	**0.60(0.55,0.64)**
Network 3: Transformer-based models						
3D VIT	0.62(0.58,0.65)	0.59(0.44,0.66)	0.55(0.48,0.60)	0.57(0.54,0.60)	0.57(0.48,0.63)	0.54(0.48,0.59)
3D SwinUNETR	0.59(0.44,0.62)	0.57(0.42,0.64)	0.57(0.47,0.62)	0.53(0.50,0.56)	0.55(0.48,0.62)	0.55(0.50,0.60)
3D MaxVit	0.69(0.66,0.73)	0.62(0.55,0.69)	0.64(0.59,0.69)	0.62(0.59,0.65)	0.60(0.54,0.67)	0.60(0.55,0.65)
**CNN-Transformer(ours)**	**0.72(0.69,0.75)**	**0.68(0.62,0.73)**	**0.68(0.63,0.73)**	**0.65(0.62,0.67)**	**0.61(0.55,0.68)**	**0.64(0.60,0.68)**

Note: The reported values represent Harrell's concordance index (C-index) with a 95% confidence interval (CI). OS, overall survival. PFS, progression-free survival. CNN, convolutional neural network.

6

7

Supplementary Table 3. Ablation analysis to optimize Network 1 to Network 3 in overall survival and progression-free survival.

Input images	Network1			Network2			Network3		
	**Training**	**Validation**	**Testing**	**Training**	**Validation**	**Testing**	**Training**	**Validation**	**Testing**
**overall survival**									
Arterial	0.65	0.61	0.59	0.57	0.52	0.55	0.62	0.58	0.62
Portal vein	0.63	0.54	0.58	0.63	0.59	0.57	0.63	0.60	0.61
Delayed	0.67	0.61	0.51	0.58	0.58	0.55	0.62	0.58	0.59
A+V	0.60	0.59	0.55	0.57	0.55	0.52	0.64	0.59	0.62
A+D	0.75	0.59	0.59	0.60	0.58	0.58	0.61	0.59	0.61
V+D	0.61	0.56	0.57	0.59	0.58	0.58	0.64	0.60	0.61
A+V+D	0.62	0.63	0.61	0.66	0.62	0.60	0.65	0.61	0.64
**progression-free survival**									
Arterial	0.69	0.61	0.62	0.62	0.57	0.58	0.66	0.60	0.66
Portal vein	0.63	0.55	0.54	0.66	0.61	0.61	0.67	0.64	0.63
Delayed	0.74	0.64	0.61	0.63	0.62	0.62	0.67	0.61	0.64
A+V	0.70	0.61	0.60	0.64	0.62	0.60	0.69	0.63	0.66
A+D	0.67	0.59	0.59	0.64	0.61	0.62	0.67	0.63	0.65
V+D	0.67	0.60	0.60	0.63	0.60	0.63	0.70	0.63	0.65
A+V+D	0.68	0.65	0.65	0.74	0.65	0.64	0.72	0.68	0.68

A, Arterial Phase; V, Portal Venous Phase; D, Delayed Phase

Supplementary Table 4. Ablation study evaluating different combinations of individual network models.

Ensemble	Network1	Network2	Network3	OS			PFS		
				Training	Validation	Testing	Training	Validation	Testing
**Single model**	√	X	X	0.68	0.65	0.65	0.62	0.63	0.61
	X	√	X	0.74	0.65	0.64	0.66	0.62	0.60
	X	X	√	0.72	0.68	0.68	0.65	0.61	0.64
**Two models**	√	√	X	0.75	0.67	0.66	0.68	0.64	0.62
	X	√	√	0.75	0.68	0.67	0.69	0.63	0.64
	√	X	√	0.73	0.69	0.68	0.67	0.63	0.65
**All**	√	√	√	**0.76**	**0.70**	**0.69**	**0.69**	**0.64**	**0.66**

Note: The reported values represent Harrell's concordance index (C-index). OS, overall survival. PFS, progression-free survival.

Supplementary Table 5. Radiomic features selected in the pre-treatment CT images on overall survival and progression-free survival.

Feature name	Abbreviation
**overall survival**	
GLRLM_GrayLevelNonUniformity(Liver,A)	L_A_glrlmGLNU
GLSZM_LargeAreaLowGrayLevelEmphasis(Liver,A)	L_A_ glszmLALGLE
FirstOrder_InterquartileRange(Liver,V)	L_V_ firstoderIR
GLDM_SmallDependenceLowGrayLevelEmphasis (Liver,V)	L_V_ gldmSDLGLE
GLSZM_LargeAreaHighGrayLevelEmphasis(Liver,V)	L_V_glszmLAHGLE
FirstOrder_Skewness(Liver,D)	L_D_firstoderS
FirstOrder _Minimum(Tumor,V)	T_V_firstoderM
GLSZM_SizeZoneNonUniformityNormalized(Tumor,V)	T_V_glszmSZNUN
GLSZM_ZoneVariance(Tumor,D)	T_D_glszmZV
**progression-free survival**	
GLRLM_GrayLevelNonUniformity(Liver,A)	L_A_glrlmGLNU
GLSZM_LargeAreaHighGrayLevelEmphasis(Liver,V)	L_V_glszmLAHGLE
GLSZM_ZoneEntropy(Tumor,A)	T_A_glszmZE
GLSZM_ZoneVariance(Tumor,A)	T_A_glszmZV
GLSZM_SizeZoneNonUniformityNormalized(Tumor,V)	T_V_glszmSZNUN
GLCM_Imc1(Tumor,D)	L_D_firstoderS

Note. liver and tumor denote radiomic features extracted on the whole liver parenchyma and primary tumor, respectively. GLRLM , gray level run length matrix; GLSZM , gray-level size zone matrix; GLDM , gray level dependence matrix; GLCM, gray-level co-occurrence matrix; A, arterial phase; V, portal vein phase; D, delayed phase.

Supplementary Table 6. Univariable and multivariable analyses of variables for overall survival in the discovery cohort and testing cohort.

Variables	Discovery(n= 650)				Testing(n= 209)			
	Univariable		Multivariable		Univariable		Multivariable	
	HR(95%CI)	*p* value	HR(95%CI)	*p* value	HR(95%CI)	*p* value	HR(95%CI)	*p* value
Ensemble-DL	3.37(2.91-3.91)	<.001	3.06(2.57-3.63)	<.001	3.51(2.33-5.28)	<.001	3.38(2.11-5.41)	<.001
Age (<= 50years vs > 50years)	0.65(0.52-0.81)	<.001	0.99(0.78-1.25)	.918	1.01(0.60-1.69)	.984	2.62(1.44-4.78)	.002
Sex (male vs female)	0.90(0.66-1.23)	.515	0.74(0.54-1.03)	.071	0.95(0.52-1.74)	.874	1.19(0.61-2.32)	.601
HBV infection(absent vs present)	1.04(0.79-1.36)	.800	0.87(0.59-1.28)	.469	0.78(0.49-1.22)	.269	0.91(0.54-1.55)	.731
BCLC stage (B vs C)	0.28(0.16-0.46)	<.001	0.64(0.32-1.28)	.209	0.43(0.21-0.87)	.02	1.57(0.49-4.98)	.446
Child_Pugh (A vs B)	1.57(1.22-2.03)	<.001	1.18(0.90-1.55)	.221	2.93(1.82-4.73)	<.001	2.36(1.38-4.04)	.002
Cirrhosis (absent vs present)	0.77(0.59-0.99)	.040	0.70(0.49-1.00)	.050	0.66(0.41-1.06)	.088	0.84(0.49-1.46)	.538
AFP level(<= 400 vs > 400ng/mL)	1.64(1.33-2.02)	<.001	1.37(1.11-1.71)	.004	1.55(1.07-2.24)	.019	1.01(0.67-1.54)	.950
Line of ICIs therapy (1 vs >= 2)	1.41(1.07-1.86)	.014	1.10(0.81-1.49)	.543	0.59(0.27-1.26)	.173	0.83(0.36-1.9)	.604
Previous local therapy(absent vs present)	1.08(0.89-1.31)	.411	1.17(0.95-1.44)	.128	0.79(0.53-1.17)	.236	0.85(0.57-1.26)	.416
PVTT (absent vs present)	2.42(1.72-3.41)	<.001	0.95(0.58-1.55)	.83	2.14(1.24-3.69)	.006	1.57(0.62-4.01)	.343
LungMet (absent vs present)	2.01(1.6-2.53)	<.001	1.41(1.11-1.79)	.005	2.02(1.31-3.12)	.001	2.61(1.55-4.37)	<.001
BoneMet (absent vs present)	2.37(1.55-3.63)	<.001	2.21(1.41-3.45)	.001	0.75(0.10-5.39)	.776	2.91(0.33-25.69)	.335
LNMet (absent vs present)	1.89(1.52-2.36)	<.001	1.49(1.18-1.88)	.001	1.92(1.34-2.83)	<.001	1.42(0.94-2.13)	.094
Up to Seven (<= 7 vs > 7)	2.13(1.72-2.66)	<.001	1.72(1.37-2.16)	<.001	1.81(1.24-2.63)	.002	1.35(0.90-2.02)	.147
Maximum tumor diameter(<= 5 vs > 5cm)	2.18(1.64-2.91)	<.001	1.02(0.71-1.46)	.909	2.54(1.51-4.27)	<.001	1.42(0.76-2.68)	.273
ECOG PS(0 vs 1)	1.01(0.82-1.24)	.931	1.05(0.85-1.31)	.639	2.87(1.98-4.15)	<.001	1.99(1.3-3.06)	.002

BCLC = Barcelona Clinic Liver Cancer, AFP = alpha-fetoprotein, PVTT = portal vein tumor thrombus, LNMet = lymph node metastasis, ECOG PS = Eastern Cooperative Oncology Group Performance Status, ICIs = immune checkpoint inhibitors, HR= hazard ratios.

Supplementary Table 7. Univariable and multivariable analyses of variables for Progression-free survival in the discovery cohort and testing cohort.

Variables	Discovery(n= 650)				Testing(n= 209)			
	Univariable		Multivariable		Univariable		Multivariable	
	HR(95%CI)	*p* value	HR(95%CI)	*p* value	HR(95%CI)	*p* value	HR(95%CI)	*p* value
Ensemble-DL	1.97(1.76-2.20)	<.001	1.89(1.66-2.14)	<.001	1.87(1.53-2.29)	<.001	1.81(1.42-2.31)	<.001
Age (<= 50years vs > 50years)	0.76(0.62-0.93)	.008	0.94(0.77-1.16)	.576	0.81(0.54-1.22)	.320	1.44(0.92-2.27)	.115
Sex (male vs female)	0.96(0.75-1.24)	.775	0.88(0.68-1.44)	.340	1.38(0.89-2.15)	.154	1.52(0.95-2.45)	.080
HBV infection (absent vs present)	1.01(0.80-1.27)	.958	0.94(0.70-1.27)	.680	0.91(0.62-1.34)	.628	1.21(0.78-1.87)	.393
BCLC stage (B vs C)	0.47(0.33-0.67)	<.001	0.96(0.58-1.61)	.885	0.69(0.42-1.13)	.140	1.42(0.63-3.22)	.403
Child_Pugh (A vs B)	1.42(1.13-1.77)	.002	1.26(1.00-1.58)	.050	1.79(1.15-2.78)	.010	1.36(0.83-2.23)	.220
Cirrhosis (absent vs present)	0.86(0.70-1.06)	.167	0.82(0.63-1.07)	.150	0.92(0.64-1.32)	.655	1.32(0.87-2.02)	.195
AFP level (<= 400 vs > 400ng/mL)	1.50(1.26-1.80)	<.001	1.19(0.99-1.44)	.063	1.25(0.92-1.71)	.153	1.09(0.76-1.55)	.641
Line of ICIs therapy (1 vs >= 2)	1.43(1.12-1.83)	.005	1.47(1.12-1.91)	.005	0.89(0.51-1.54)	.672	1.06(0.89-1.91)	.842
Previous local therapy (absent vs present)	1.08(0.92-1.27)	.367	1.08(0.91-1.28)	.383	0.83(0.60-1.14)	.253	1.02(0.73-1.42)	.909
PVTT (absent vs present)	1.76(1.36-2.29)	<.001	1.03(0.69-1.55)	.886	1.51(1.01-2.28)	.042	1.52(0.76-3.06)	.238
Lunged (absent vs present)	1.77(1.44-2.17)	<.001	1.52(1.23-1.89)	<.001	1.54(1.04-2.28)	.031	1.78(1.13-2.82)	.013
BoneMet (absent vs present)	1.72(1.16-2.55)	.007	1.28(0.84-1.95)	.243	0.49(0.07-3.51)	.479	1.50(0.19-12.04)	.704
LNMet (absent vs present)	1.60(1.33-1.93)	<.001	1.33(1.08-1.62)	.006	1.72(1.25-2.35)	<.001	1.30(0.91-1.85)	.148
Up to Seven (<= 7 vs > 7)	1.92(1.60-2.30)	<.001	1.77(1.47-2.14)	<.001	1.75(1.29-2.38)	<.001	1.66(1.18-2.34)	.004
Maximum tumor diameter (<= 5 vs > 5cm)	1.69(1.35-2.11)	<.001	0.98(0.73-1.31)	.890	1.66(1.15-2.41)	.010	1.04(0.65-1.65)	.884
ECOG PS(0 vs 1)	1.02(0.86-1.22)	.819	1.01(0.84-1.21)	.889	2.18(1.60-2.97)	<.001	1.80(1.27-2.55)	.001

BCLC = Barcelona Clinic Liver Cancer, AFP = alpha-fetoprotein, PVTT = portal vein tumor thrombus, LNMet = lymph node metastasis, ECOG PS = Eastern Cooperative Oncology Group Performance Status, ICIs = immune checkpoint inhibitors, HR= hazard ratios.

12

Supplementary Table 8. Agents administration protocol

Agents	Targets	Administration
**Anti-PD-(L)1 agents administration**		
Atezolizumab, Tecentriq®, F. Hoffmann-La Roche AG, Basel, Switzerland	PD-L1	1200 mg, ivgtt, q3w
Camrelizumab, AiRuiKa®, Jiangsu Hengrui Medicine Co. Ltd, Suzhou, China	PD-1	200 mg or 3mg/kg, ivgtt, q3w
Pembrolizumab, Keytruda®, Merck Sharp& Dohme Corp., Kenilworth, N.J., U.S.A.	PD-1	200 mg, ivgtt, q3w
Sintilimab, Tyvyt®, Innovent Biologics, Inc., Suzhou, China	PD-1	200 mg, ivgtt, q3w
Tislelizumab, Baize'an®, BeiGene Ltd., Beijing, China	PD-1	200 mg, ivgtt, q3w
**Molecular targeted agents administration**		
Apatinib, Aitan®, Jiangsu Hengrui Medicine Co. Ltd, Lianyungang, China	VEGFR2	250 mg, po, qd
Donafenib, Zepsun®, Suzhou Zelgen Biopharmaceuticals Co, Ltd., Suzhou, China	VEGFR,PDGFR, Raf/MEK/ERK kinase	200 mg BID
Bevacizumab, Avastin®, F. Hoffmann-La Roche AG, Basel, Switzerland	VEGFA	15 mg/kg, ivgtt, q3w
Lenvatinib, Lenvanix®, Eisai Inc., Japan	VEGFR1–VEGFR3, PDGFR, FGFR1–FGFR4, RET	8 mg, po, qd (for bodyweight < 60 kg) or 12 mg, po, qd (for bodyweight ≥ 60 kg)
Sorafenib, Nexavar®, Bayer AG Kaiser-Wilhelm-Allee, Leverkusen, Germany	VEGFR1–VEGFR3, PDGFR, RAF kinase, KIT receptor	400 mg, po, bid

2. Supplementary Figures

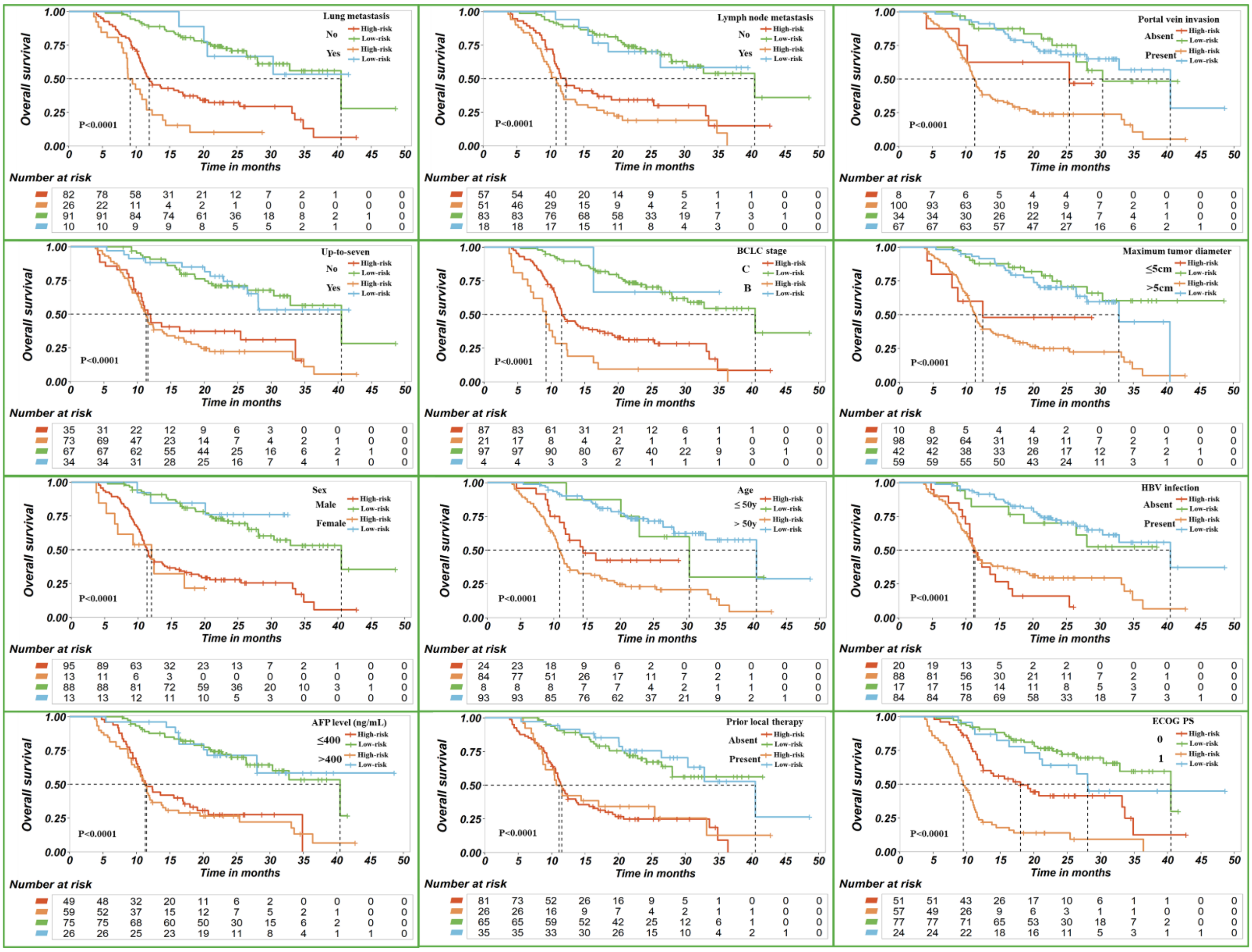

Supplementary Fig. 1. Subgroup analyses of MMF system for overall survival in testing cohort. Columns depict: (1) lung/lymph node metastasis, portal vein invasion; (2) tumor number (up to 7), BCLC stage, max diameter; (3) sex, age, HBV infection; (4) AFP, prior local therapy, ECOG performance status. MMF, multimodal fusion. P values were calculated using a two-sided log-rank test.

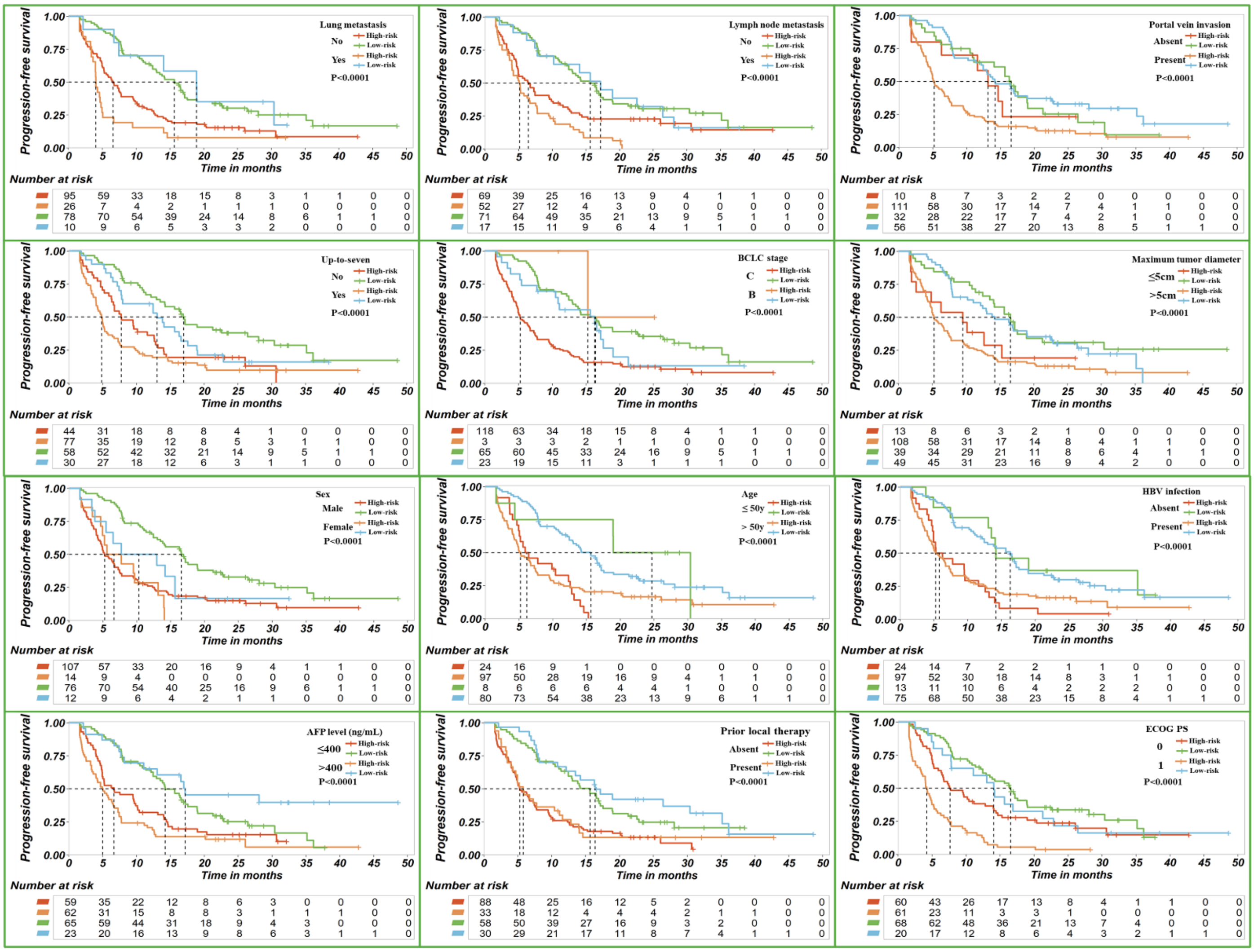

Supplementary Fig. 2. Subgroup analyses of MMF system for progression-free survival in testing cohort. Columns depict: (1) lung/lymph node metastasis, portal vein invasion; (2) tumor number (up to 7), BCLC stage, max diameter; (3) sex, age, HBV infection; (4) AFP, prior local therapy, ECOG performance status. MMF, multimodal fusion. P values were calculated using a two-sided log-rank test.

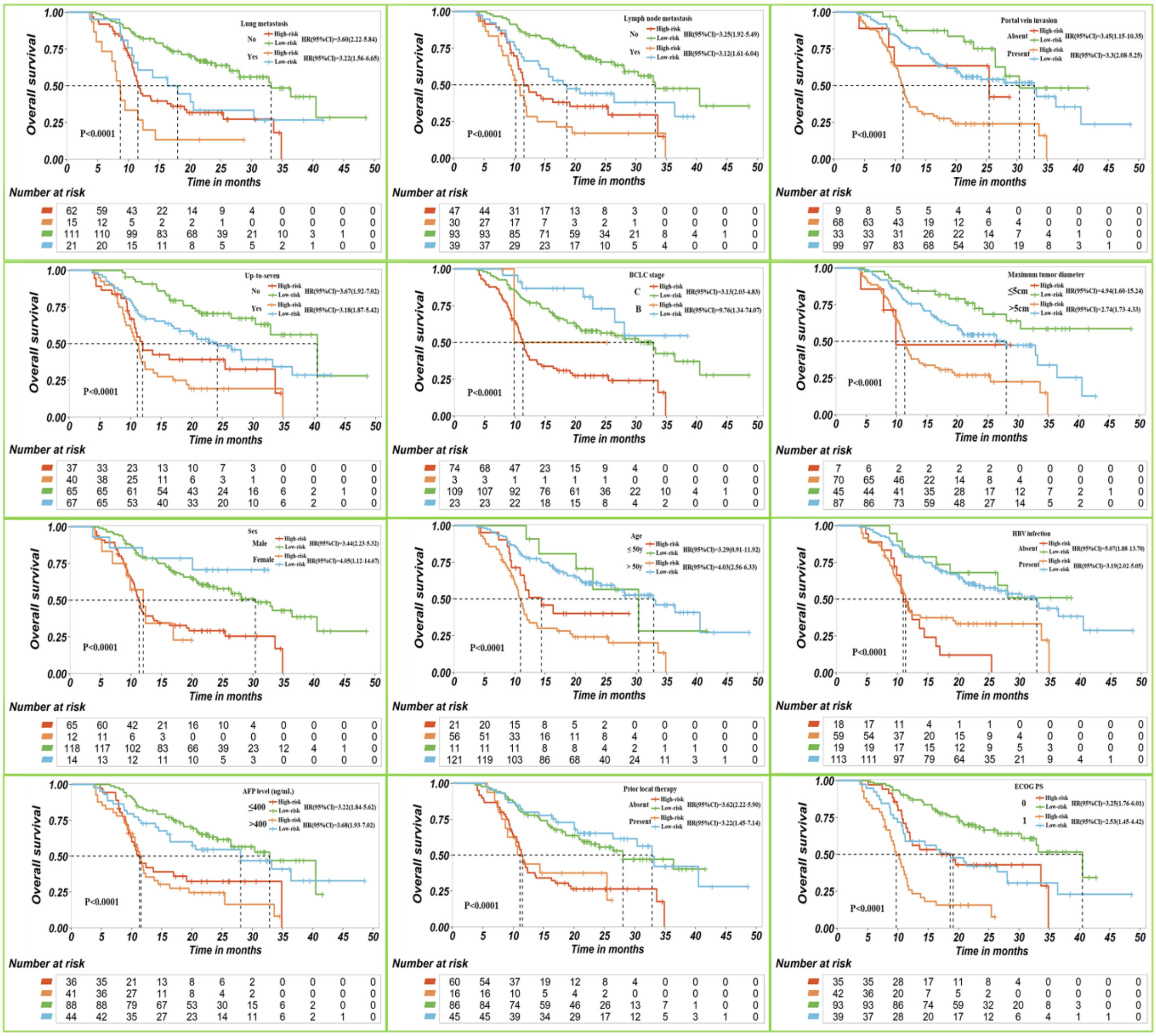

Supplementary Fig. 3. Subgroup analyses of Ensemble-DL model for overall survival in testing cohort. Columns depict: (1) lung/lymph node metastasis, portal vein invasion; (2) tumor number (up to 7), BCLC stage, max diameter; (3) sex, age, HBV infection; (4) AFP, prior local therapy, ECOG performance status. Ensemble-DL, ensemble deep learning. P values were calculated using a two-sided log-rank test.

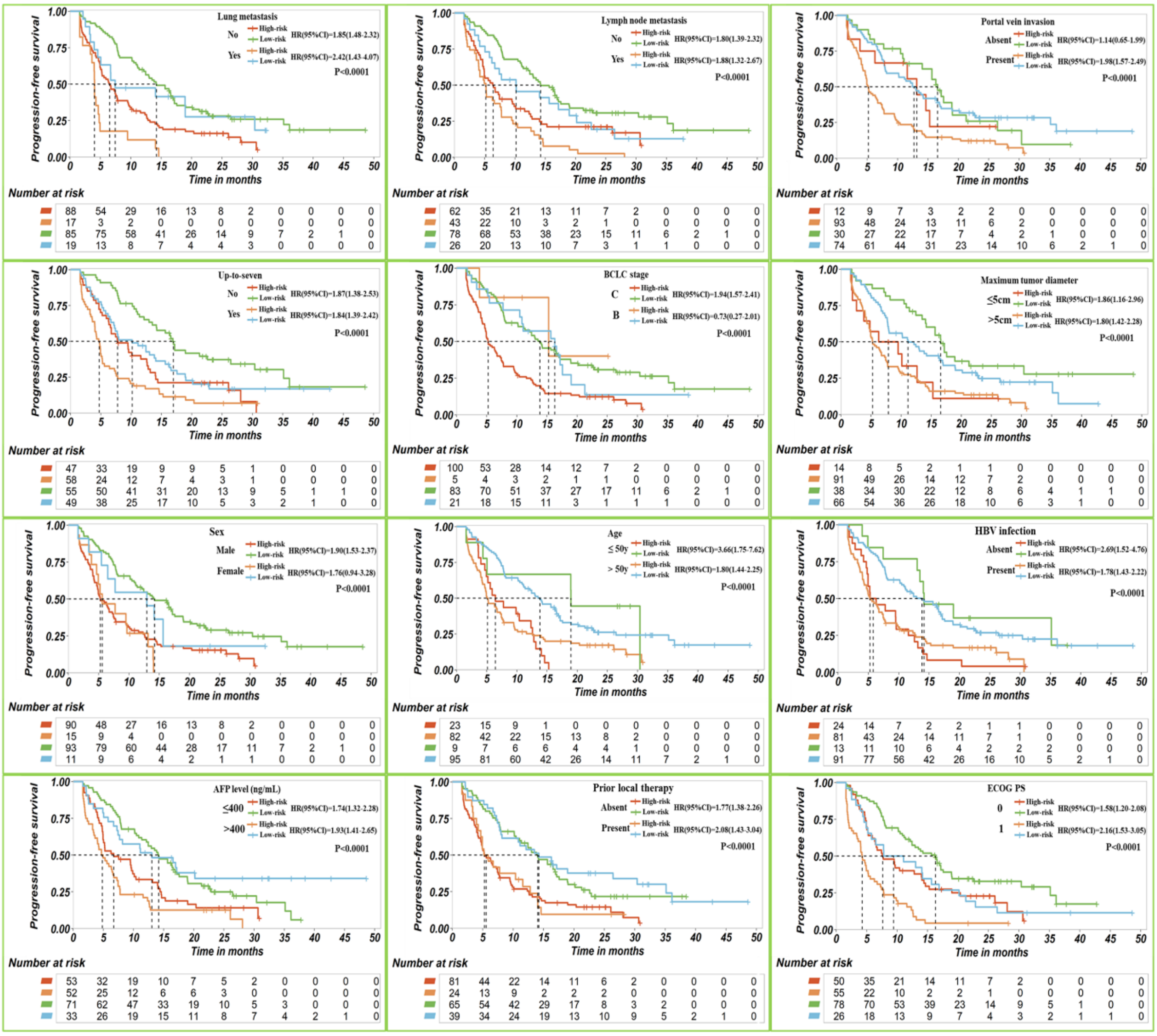

Supplementary Fig. 4. Subgroup analyses of Ensemble-DL model for progression-free survival in testing cohort. Columns depict: (1) lung/lymph node metastasis, portal vein invasion; (2) tumor number (up to seven), BCLC stage, max diameter; (3) sex, age, HBV infection; (4) AFP, prior local therapy, ECOG performance status. Ensemble-DL, ensemble deep learning. P values were calculated using a two-sided log-rank test.

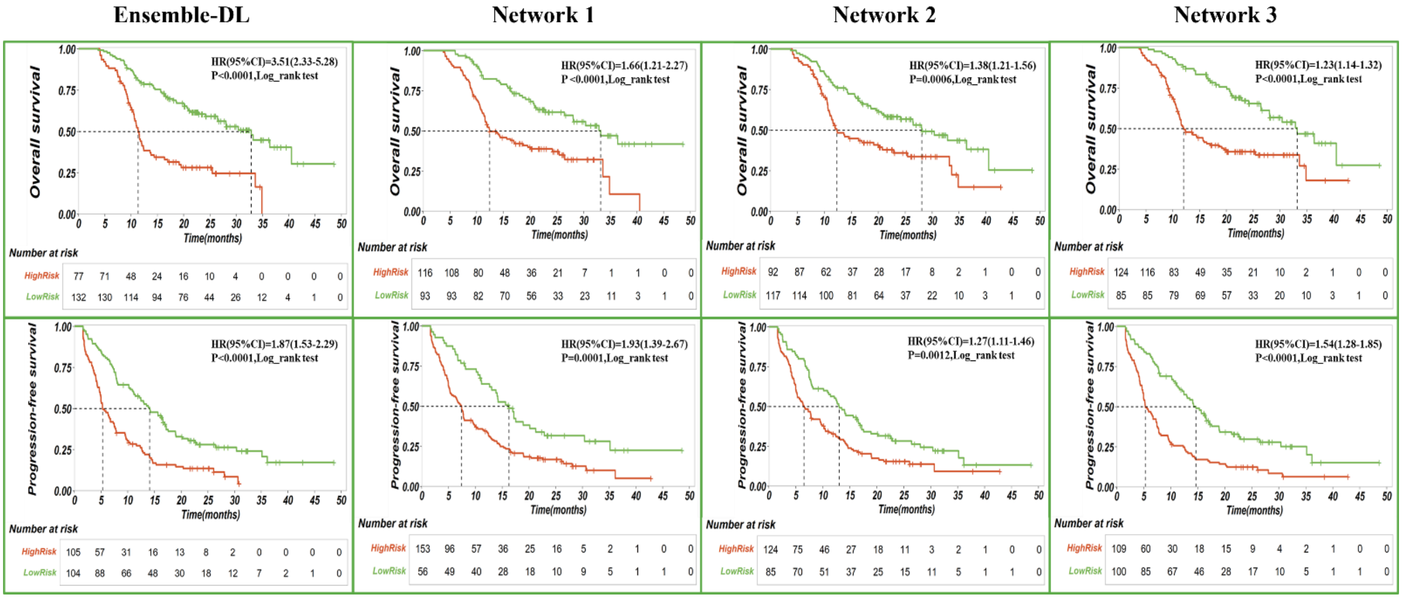

Supplementary Fig. 5. Stratified Kaplan-Meier curves for OS and PFS in the testing cohorts with Ensemble-DL and individual deep learning models. OS, overall survival. PFS, progression-free survival. Ensemble-DL, ensemble deep learning. HR, hazard ratio. P values were calculated using a two-sided log-rank test.

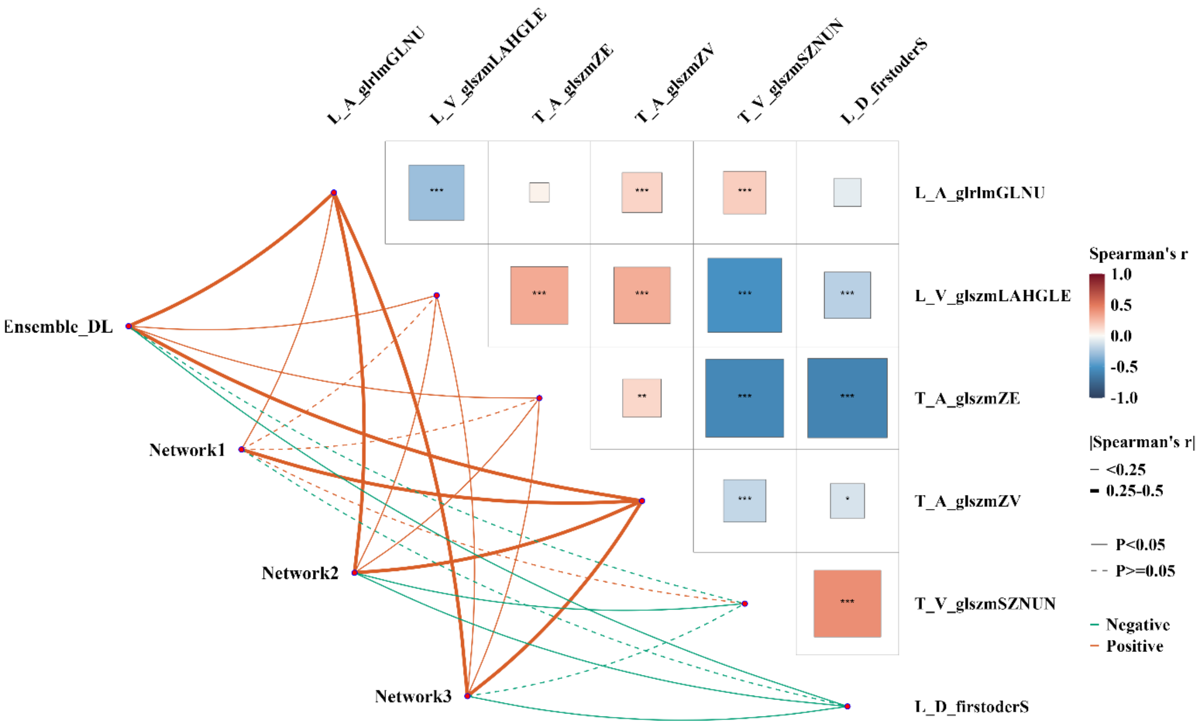

Supplementary Fig. 6. Deep Learning-Radiomics Correlation: Associations between deep learning signatures and radiomics features related to progression-free survival.

3. Supplementary Information 1: CT imaging Protocol, Administration of ICIs and Assessments

CT Imaging Protocol

All patients underwent multiphase contrast-enhanced abdominal CT scans performed using a variety of CT systems, including the GE LightSpeed VCT, GE Revolution CT, PHILIPS Brilliance 64, GE Discovery CT750, SIEMENS SOMATOM Definition Flash, or GE Optima CT680. Imaging parameters included a tube current of 260-380 mA, tube voltage of 100-120 kV, field of view of 350 × 350 mm to 500 × 500 mm, matrix size of 512 × 512, and slice thickness of 0.625 mm to 5 mm. Nonionic iodinated contrast agents (Iohexol, Omnipaque 300, GE Healthcare, Shanghai, China) were administered intravenously at a dose of 1.5 mL/kg body weight at a 3.0-4.0 mL/sec rate. Arterial phase imaging was acquired using bolus tracking to achieve 100 HU aortic enhancement. Subsequent portal venous and delayed phases were obtained at 60-75 seconds and 150-180 seconds post-contrast.

Administration of Immune Checkpoint Inhibitors and Assessments

Patients received various immune checkpoint inhibitors (ICIs) and molecular-targeted agents (TKIs or anti-vascular endothelial growth factor agents) according to Chinese government guidelines and drug availability (**Supplementary Table 8**). Among the 859 patients with unresectable HCC, 47.8% (410/859) received the standard camrelizumab and apatinib regimen, with 49.8% (324/650) in the discovery cohort and 41.1% (86/209) in the external test cohort. Other treatment regimens included sintilimab plus lenvatinib in 12.0% (103/859), tislelizumab plus lenvatinib in 10.4% (89/859), sintilimab plus bevacizumab in 4.8% (41/859), atezolizumab plus bevacizumab in 5.0% (43/859), pembrolizumab plus lenvatinib in 3.8% (33/859), and ICIs combined with other molecular targeted therapies (such as sorafenib, donafenib, etc.) in 16.3% (140/859). Each ICI was administered at standard doses and frequencies until disease progression or intolerable immune-related adverse reactions occurred. Typical doses of various molecular-targeted agents were also administered. Oral molecular-targeted medications were given within two weeks before or after administering ICIs, while bevacizumab (an anti-VEGF agent) was administered concurrently with ICIs.

mRECIST defines tumor response as follows:

Complete response (CR): Disappearance of all target lesion arterial enhancement. Partial response (PR): ≥30% decrease in the sum of the longest diameter of target lesions compared to baseline. Progressive disease (PD): ≥20% increase in the sum of the diameters of target lesions (arterial phase enhancement) from the smallest sum observed since treatment started or the appearance of new lesions. Stable disease (SD): Neither CR, PR, nor PD. Patients were divided into responders (CR/PR) and non-responders (SD/PD) based on the objective response rate (ORR) as defined by mRECIST criteria. Then we construct an mRECIST prognostic model based on the first follow-up CT mRECIST assessment results using Cox proportional hazards regression.

4. Supplementary Information 2: Structure of three deep learning networks

Network 1 (EfficientNet B1 Model): This subnetwork uses the EfficientNet B1 convolutional neural network (CNN) for supervised learning. It starts with a 3D convolutional layer using 32 filters, a 3x3x3 kernel, and a stride of 2 in each dimension, followed by padding and 3D batch normalization layer with 32 features. The backbone network includes seven sequential blocks, each containing a varying number of MBConv modules. Each MBConv module consists of depthwise and pointwise convolutions, along with squeeze-and-excitation (SE) blocks for channel attention. These modules, which have varying numbers of input and output channels based on their network position, learn hierarchical features at different scales. EfficientNet model employs a multi-task learning scheme with separate output channels for predicting overall survival (OS) and progression-free survival (PFS) using a Cox proportional hazards model for survival loss and binary cross-entropy for clinical variables, while inferring additional risk factors through auxiliary channels. A heteroscedastic uncertainty-based multi-task loss function optimizes these losses simultaneously without manual weight tuning [1].

Network 2 (Semi-supervised hybrid model): This subnetwork employs a two-stage approach, consisting of an encoder, a bottleneck, a decoder, and a survival network (DeepSurv)[2] for predicting survival-related outcomes. Initially, unsupervised feature learning is conducted using an autoencoder, which reconstructs the original CT data and trains the encoder to effectively compress the dimensionality of the CT input. The encoder comprises four Residual Unit blocks, each reducing spatial dimensions of the input while increasing channel numbers. The first Residual Unit takes a 3-channel input and applies a series of convolutional layers with 32 filters, followed by instance normalization, dropout, and PReLU activation. The residual connection in this block also applies a convolutional layer to match the dimensions of the output. The subsequent Residual Unit blocks follow a similar structure, with increasing numbers of filters (64, 128, 256) and decreasing spatial dimensions. Subsequently, compressed features from the bottleneck layer are fed into the DeepSurv model for supervised survival prediction.

The bottleneck contains two Residual Unit blocks that maintain the spatial dimensions and channel numbers from the encoder's output, applying convolutional layers, instance normalization, dropout, and PReLU activation with an identity residual connection. The decoder consists of four blocks that upsample spatial dimensions and decrease channel numbers, each applying a transposed convolution followed by a Residual Unit with instance normalization, dropout, and PReLU activation, and an identity residual connection. The final decoder block outputs a single-channel result matching the input's spatial dimensions.

The DeepSurv network[2], a deep learning-based survival analysis model, extends the Cox proportional hazards framework by incorporating non-linear feature representations. In our implementation, the DeepSurv architecture processes the encoder's final output (256 channels) through convolutional layers with increasing filters (256, 512, 1024),

batch normalization, PReLU activation, and dropout. The output is then flattened and passed through four separate fully connected layers for predicting survival-related outcomes. Similar to the first network, a multi-task learning scheme optimizes losses for PFS and OS, reconstruction loss, and auxiliary losses associated with conventional risk variables.

Network 3 (CNN-Transformer Model): This subnetwork adopts a CNN-Transformer architecture based on multi-plane and multi-slice Transformer networks. The model comprises five main components: a 3D Convolutional Block, a Multi-Plane/Slice Token Extraction Block, 2D Convolutional Block, Embedding Block, and a Transformer encoder.

1. 3D Convolutional Block: Extracts native 3D features from the input data. The 3D convolutional backbone consists of two 3D convolutional layers with ReLU activations. The first convolutional layer employs 32 filters with a 5x5x5 kernel and 2-pixel padding. The subsequent convolutional layer maintains the same filter count, kernel size, and padding.
2. Multi-Plane/Slice Token Extraction Block: Extracts features from different spatial and temporal planes, projecting them into a lower-dimensional space to generate tokens from the 3D features across multiple planes (axial, coronal, sagittal) and slices. It then interacts each slice with its corresponding position in the entire 3D volume using element-wise multiplication. It first applies global average pooling to reduce the spatial dimensions. Then, a two-layer fully connected network with ReLU activations is used for projection.
3. 2D Convolutional Block: Utilizes a pre-trained ResNet50 model for global average pooling and converts the features into embedding vectors through a non-linear projection layer.
4. Embedding Block: Combines embedding vectors from the previous block with positional and plane embedding vectors.
5. The Transformer network uses positional and plane embeddings to capture interrelationships across multiple image slices and planes. It outputs a final feature vector of dimension 756. The network architecture comprises 12 layers, each utilizing a hidden size and feed-forward network size of 768 with 8 attention heads. Each encoder block integrates multi-head attention and feed-forward sub-layers to capture long-range dependencies within the data. The feed-forward network applies a fully connected network to each position in the sequence independently.

Each deep learning network was implemented using the PyTorch framework and trained for 250 epochs on an NVIDIA RTX 3090 GPU. A combined loss function, incorporating Cox proportional hazards and binary cross-entropy losses, was used, with variance-based weighting for effective optimization. Batch sizes were set at 16 for Networks 1 and 2, and 4 for Network 3. Training employed the Adam optimizer with a learning rate of 0.0001, incorporating data augmentation, dropout, learning rate decay, and L1 regularization to reduce overfitting. Model hyperparameters were fine-tuned on the training cohort to achieve optimal performance in the validation cohort.

5. Supplementary References

1. Kendall, A. & Cipolla, R. Multi-Task Learning Using Uncertainty to Weigh Losses for Scene Geometry and Semantics. (2018).
2. Katzman, J.L., *et al.* DeepSurv: personalized treatment recommender system using a Cox proportional hazards deep neural network. *BMC Med Res Methodol* **18**, 24 (2018).
